# LigTMap: ligand and structure-based target identification and activity prediction for small molecular compounds

**DOI:** 10.1186/s13321-021-00523-1

**Published:** 2021-06-10

**Authors:** Faraz Shaikh, Hio Kuan Tai, Nirali Desai, Shirley W. I. Siu

**Affiliations:** 1 Department of Computer and Information Science, Faculty of Science and Technology, University of Macau, Avenida da Universidade, Taipa, Macau China; 2 Division of Biological and Life Sciences, Ahmedabad University, Ahmedabad, India

**Keywords:** Target prediction, Binding affinity prediction, Fingerprint similarity, Binding interaction fingerprint, Inverse docking, Drug repurposing, PSOVina, Random forest

## Abstract

Target prediction is a crucial step in modern drug discovery. However, existing experimental approaches to target prediction are time-consuming and costly. Here, we introduce LigTMap, an online server with a fully automated workflow that can identify protein targets of chemical compounds among 17 classes of therapeutic proteins extracted from the PDBbind database. It combines ligand similarity search with docking and binding similarity analysis to predict putative targets. In the validation experiment of 1251 compounds, targets were successfully predicted for more than 70% of the compounds within the top-10 list. The performance of LigTMap is comparable to the current best servers SwissTargetPrediction and SEA. When testing with our newly compiled compounds from recent literature, we get improved top 10 success rate (66% ours vs. 60% SwissTargetPrediction and 64% SEA) and similar top 1 success rate (45% ours vs. 51% SwissTargetPrediction and 41% SEA). LigTMap directly provides ligand docking structures in PDB format, so that the results are ready for further structural studies in computer-aided drug design and drug repurposing projects. The LigTMap web server is freely accessible at https://cbbio.online/LigTMap. The source code is released on GitHub (https://github.com/ShirleyWISiu/LigTMap) under the BSD 3-Clause License to encourage re-use and further developments.

**Supplementary Information:**

The online version contains supplementary material available at 10.1186/s13321-021-00523-1.

## Introduction

In recent years, the number of small natural and synthetic molecules, both real and virtual, has significantly increased [1]. One way to evaluate their potential for therapeutic applications is to identify their molecular targets related to diseases. Similarly, compared with traditional methods, finding new targets for existing drugs, that is, drug repurposing, can disclose new clinical applications of known drugs in a shorter time and at a lower cost [2]. On the other hand, the newly discovered molecular targets of existing drugs may implicate the potential side effects and toxicity of the drug, so efforts should be made to improve the safety of these drugs [3]. Despite technological advances, experimental methods to target identification remain laborious, expensive, and sometimes unsuccessful. Moreover, initial hypotheses on the potential target are typically required as the basis for the design of effective biochemical and genetic interaction experiments [4].

Over the years, various in silico approaches have been developed to provide solutions to the target prediction problem [5]. Additional file 1: Table S1 presents a list of some of these computational target prediction methods, highlighting their methodological strategies, employed datasets, and availability of online servers. These approaches can be broadly classified into three groups: ligand-based, structure-based, and hybrid [6, 7]. The central notion of ligand-based approaches is that chemically similar compounds exhibit analogous biological activities [8]. Thus, ligand-based methods extract chemical features of molecules using fingerprint algorithms to compare the similarities between the query compounds and the ligands with known activities [8]. Despite their simplicity, with prior knowledge of ligands and their targets, ligand-based methods are effective and fast. Nevertheless, their domains of application are limited by the available chemical and biological data [9]. Furthermore, it is not straightforward to define cutoffs for chemical similarity measures, as they strongly depend on the fingerprints used and classes of the compounds under study [10]. Examples of ligand-based methods include SEA [11], SuperPred [12], PASS [13], and TarPred [14]. To enhance predictive performance, newer methods have emerged which utilize supervised (such as HitPick [15] and Target Hunter [16]) and unsupervised machine learning (ML) (such as SPiDER [17]) to improve the model precision rate. In addition, some of the earlier methods, such as ChemProt [18] and SwissTargetPrediction [19, 20] have also updated their search engines to use ML models which have shown greater effectiveness [21, 22].

On the other hand, structure-based approaches utilize the available three-dimensional (3D) structures of targets. They either apply docking to estimate the structural and chemical *fitness* of the query compound to the target or extract a set of pharmacophores from protein–ligand complexes and check whether the query compound matches well with the pharmacophores. In both cases, sufficient exploration of the ligand or protein conformational space is necessary. Consequently, structure-based approaches are costly and more time-consuming than the ligand-based methods. Several such methods have been developed including TarFisDock [23], PharmMapper [24], DRAR-CPI [25], PatchSearch [26], ACID [27], and Zhang [28]. However, few of them provide online servers, and for these servers, the number of searchable targets is limited. Finally, methods that combine both ligand and structural information, such as ChemMapper [29], can be utilized to predict more complex systems [7]. In addition to typical protein or ligand data, other biological information, including protein sequences, protein–protein and protein–ligand interactions, and disease pathways, can also be used to more reliably infer the relevant targets [30].

In the present study, a new, hybrid, fully automated target prediction workflow called LigTMap was developed to predict the molecular targets of a query compound. Here, we propose the ligand similarity search as the first step to short-list putative targets, and study the influence of different fingerprints and thresholds on effective target selection. In the second step, the binding mode of the query compound into each putative target is predicted by molecular docking, and its binding mode is compared with the binding mode of the co-crystallized ligand. The ranking of the targets is based on the combined score computed from the ligand and binding similarity scores. To assess the performance of LigTMap, we compare it with four existing servers using a set of manually curated benchmark compounds.

## Materials and methods

### Target class-specific datasets

The ligand and protein structures, as well as their experimental activity data in Ki, Kd, or IC50, were obtained from the PDBbind database (version 2017) [31]. This annually updated database has been widely used as the benchmark for comparison of protein–ligand docking programs and for assessment of scoring functions. For the purpose of target prediction, we labeled each PDB structure in the dataset with its actual target class and target name by referring to the PDB database [32] and the original literature of the structure. In total, about 6000 protein–ligand complexes were processed and classified into 17 target classes. Among the target classes, 12 are human protein targets and 5 are non-human targets that are originated from viruses or bacteria. The human protein targets include kinase, transferase, beta-secretase, hydrolase, ligase, and isomerase enzymes as well as anticoagulant, bromodomain (BRD), peroxisome, estrogen, carbonic anhydrase (CA), and diabetes, while the non-human targets include human immunodeficiency virus (HIV), hepatitis C virus (HCV), influenza, tuberculosis (TB), and *Helicobacter pylori* (*H. pylori*). Table 1 lists the 17 target-specific datasets curated in this study.

**Table 1:** The 17 target class-specific datasets used in this study

Target class	Core set			Benchmark set^a^
	Total	Training	Validation	
Human target				
Kinase	2008	1608	400	18
Transferase	559	448	111	–
Beta-secretase	309	248	61	19
Hydrolase	1196	957	239	–
Anticoagulant	264	212	52	–
Carbonic anhydrase	354	285	69	16
Ligase	89	71	18	5
Bromodomain	167	133	34	19
Isomerase	110	91	19	–
Estrogen	76	61	15	–
Peroxisome	16	13	3	–
Diabetes	99	81	18	–
Non-human target				
HIV	524	419	105	10
Tuberculosis	232	186	46	11
HCV	159	127	32	–
Influenza	99	81	18	–
* Helicobacter pylori*	52	41	11	–
Total	6313	5062	1251	98

^a^For the benchmark set, where no new suitable data were found in literature, the entries are marked as “–.” Sources of benchmark data are: Kinase [46], Ligase [47], BRD [45], CA [48], beta-secretase [49], HIV [42], and TB [50]

To prepare for the prediction workflow, each ligand in the datasets was first converted into a SMILES string using the Maestro program in Schrödinger (Schrödinger Release 2017–4, 2017). Subsequently, the 2D structural fingerprints of the evaluated ligand were generated by RDKit (RDKit: Open-source cheminformatics) employing Morgan with radius of 2 (also named as the circular fingerprint), MACCS keys, Daylight, Avalon, and 3D pharmacophore fingerprint algorithms. For each protein–ligand complex, the interaction fingerprint (IFP) was extracted using the Open Drug Discovery Toolkit (Wójcikowski et al*.*, 2015). All fingerprints were saved in the binary format to accelerate the similarity analyses that were conducted for the query ligands. To prepare for docking using PSOVina2 (Tai et al*.*, 2018), the PDB files in the datasets were converted to PDBQT format using AutoDockTools (Morris et al*.*, 2009). Protein structures were processed employing the *prepare_receptor4.py* program with the options to remove water residues and nonstandard residues, create bonds, and add hydrogens if none were already present. Ligand structures were prepared using the *prepare_ligand4.py* program with the options to repair hydrogens and merge nonpolar hydrogens and lone pairs.

For method validation, each target dataset was divided into 80% training and 20% validation. The selection of ligands for the validation set is based on random selection but each ligand was checked to ensure that the correct protein target is present in the training set. Finally, there were 5062 complexes in the training set and 1251 ligands in the validation set.

To test the performance of LigTMap independently, newly identified compounds with experimentally validated targets were searched from medicinal chemistry journals. To ensure that these compounds were not already included in our datasets nor in the datasets of the methods used in the comparative study, only works reported in 2018 and later were considered. In total, 98 compounds were obtained for 7 target classes, including kinase, ligase, BRD, CA, beta-secretase, HIV, and TB. It should be noted that the benchmark data also contained compounds for multiple targets—kinase and BRD (categorized into the kinase class) as well as TB and kinase (categorized into the TB class). However, for 10 target classes new compounds were not found in the literature, and thus, they were not included in the benchmark experiments. Using the benchmark datasets, LigTMap was compared to four state-of-the-art target prediction methods including SEA [11], SwissTargetPrediction [19, 20], SuperPred [12], and HitPick [15].

### Target prediction workflow

**Fig. 1:**
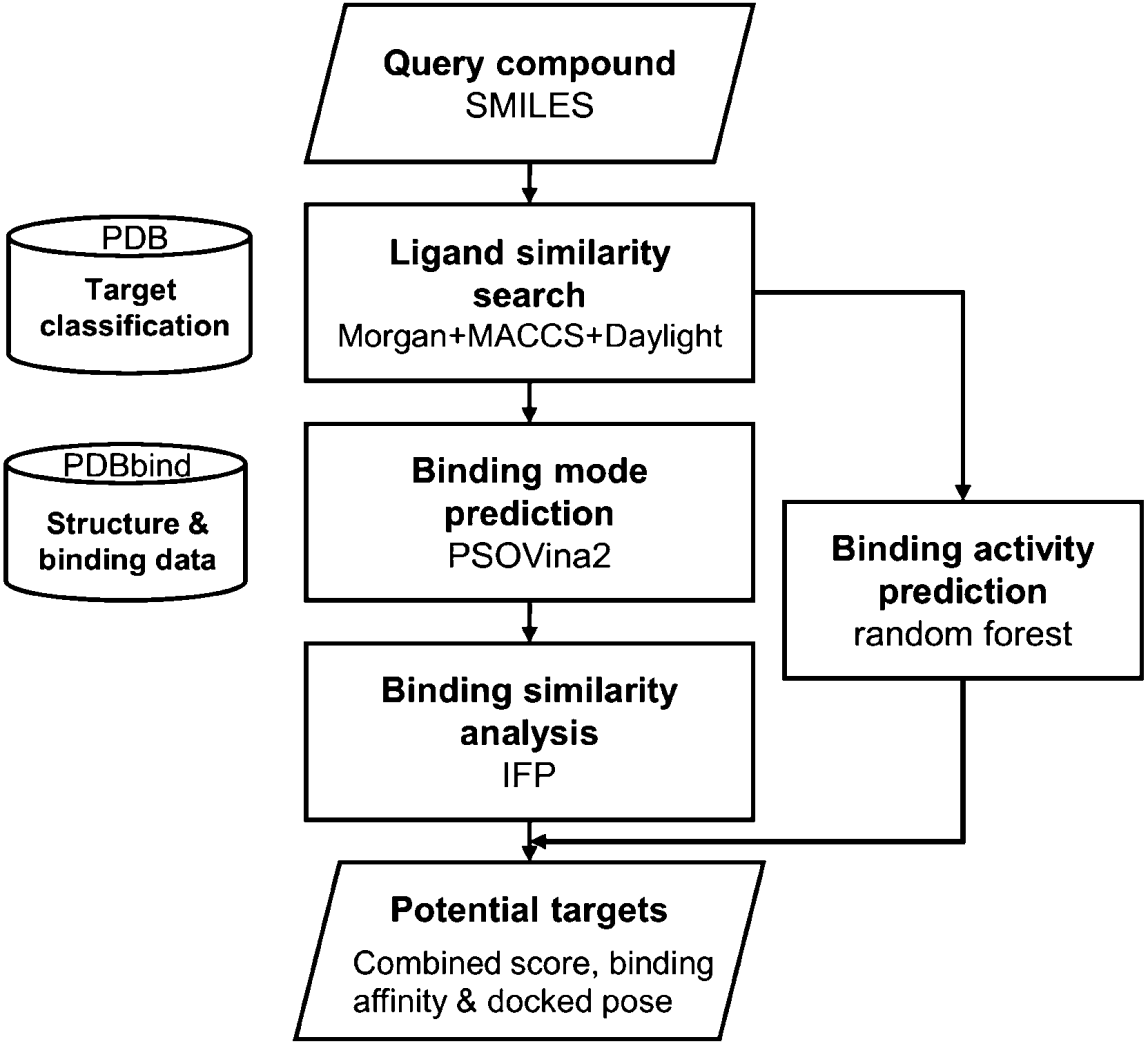
Workflow of LigTMap for target class prediction. For all classes prediction, MMD is used in the ligand similarity search, while for class-specific prediction, MM is used instead for better accuracy

The workflow of LigTMap is illustrated in Fig. 1. It consists of five steps:

1. For a query compound, a set of potential targets is selected based on fingerprint similarities to the co-crystallized ligands. Multiple fingerprints (Morgan, MACCS, Daylight, etc.) are generated, and the ligand similarity score (*T*_*L*_) is computed as an average of the fingerprint Tanimoto coefficients (T) [33]:
  1 $$T=\frac{{N}_{ab}}{{N}_{a}+{N}_{b}{- N}_{ab}}$$
  where ${N}_{a}$ and ${N}_{b}$ are the numbers of 1-bit in the fingerprints of ligand *a* and ligand *b*, respectively. ${N}_{ab}$ denotes the number of 1-bit common to both ligands. To find out which fingerprints are effective for this task, we compared the prediction performance of six fingerprints and their combinations, including Morgan, MACCS, Daylight, Atom pair, Torsion, and Pharmacophore. We will show (in the Result section) that Morgan, MACCS, and Daylight (MMD) constitute the best combination and a cutoff of 0.4 is empirically defined.
2. For each potential target, molecular docking is performed using PSOVina2 [34]. The conformation of the compound with the lowest binding free energy is taken as the optimal binding mode in the ligand-binding pocket.
3. The binding interaction fingerprint (IFP) of the compound is generated based on the predicted binding mode. The established IFP is compared with the IFP of the co-crystallized ligand using the Tanimoto coefficient to obtain the binding similarity score (*T*_*B*_).
4. For each potential target class, the compound binding activity is predicted using the class-specific random forest (RF) model and the Avalon fingerprint as the molecular descriptors.
5. Finally, all prediction results are consolidated, and the protein targets are ranked based on the combined LigTMap score that is defined as 0.7 *T*_*L*_ + 0.3 T_B_.

The parameters mentioned in this workflow were determined by testing a range of combinations using the training set and they will be discussed in the Result section.

### Performance measures for target prediction

A prediction is considered correct if the name of the predicted target matches the name of the experimental target of the test compound. Moreover, in the case of a multi-target compound, a predicted target that matches any known targets of the compound is considered a correct prediction. When testing a set of ligands, we computed the success rate of a method as the fraction of ligands in the set predicted correctly within the top *N* $\in \left\{1, 5, 10\right\}$ of the output list. In addition, for each test ligand, we computed the *recall*, *precision*, and *F1 scores* taking the top *N* targets as predicted positives (TP) and all correct targets as actual positives (TP + FN):

2 $$recall=\frac{TP}{TP+FN}$$

3 $$precision=\frac{TP}{TP+FP}$$

4 $$F1 = 2 \times \frac{{precision \times recall}}{{precision + recall}}$$

Thus, *recall* measures the proportion of correctly predicted targets among all correct targets, while *precision* indicates the proportion of correctly predicted targets within the top *N. F1* provides a single estimate that combines *recall* and *precision.*

### Ligand binding activity prediction

Once a target is identified, it is desirable to obtain an estimation of the compound binding activity toward the target. In the current method, the binding activities (pKi/Kd/IC50) are predicted by class-specific ML models. Four ML algorithms based on the Avalon fingerprint were tested, including RF, support vector machines, gradient boosted tree, and *K*-nearest neighbor algorithms. In a preliminary test of five target classes, RF exhibited superior results to other algorithms, and therefore it was selected as the final method for activity prediction of all target classes. To prevent *information leak* [35], nested cross-validation (CV) [36] was used to train and optimize the RF models. The details of the nested CV for model training and optimization are presented in Additional file 1: Section II. Methods.

## Results and discussion

LigTMap is a hybrid ligand and structure-based method for target prediction. It consists of five key steps: (1) ligand similarity search to identify potential targets and the associated target classes, (2) prediction of ligand binding modes toward the potential targets, (3) assessment of binding similarity to the co-crystallized ligand binding modes, (4) prediction of class-specific binding activity, and (5) consolidation of results and ranking.

### Selection of the fingerprint algorithm for ligand similarity search

Previous studies have shown that choosing the right fingerprint algorithm is crucial for the success of ligand-based target prediction. Furthermore, combining multiple fingerprints can improve the success rates of target prediction models [37, 38]. To establish an optimal combination of fingerprints for the ligand similarity search, we tested six different fingerprint algorithms, i.e., Morgan, MACCS, Daylight, AtomPairs, Torsion, and Pharmacophore, as well as their combinations for target prediction using the validation set. Additional file 1: Figure S2 shows the distribution of the number of ligands with correctly predicted targets, which ranked within the top 1, 5, and 10 in the output of the ligand similarity search. The figure also indicates the number of ligands whose actual target rank was below 10. Based on the results, it is clear that when the cutoff decreased from 1.0 to 0.1, the number of correctly predicted targets increased until reaching a certain cutoff value, at which the top 1/5/10 results remained relatively stable. This “optimal” range of cutoff values varied for different fingerprints. For Morgan, AtomPairs, and Torsion, it was determined at 0.1–0.3; for MACCS and Daylight, it was 0.1–0.5; and for Pharmacophore, it was 0.1–0.2. When extracting potential targets in LigTMap, if the cutoff is set too high, actual targets may be discarded too early. Conversely, if the cutoff is set too low, too many false positives are included which may result in excessive computations in the subsequent steps. Among the six fingerprints, Morgan, MACCS, and Daylight were considered as the best options. Importantly, MACCS and Daylight included correct targets with high cutoff, while Morgan predicted correct targets in top 10 for most ligands. On the other hand, AtomPairs, Torsion, and Pharmacophore exhibited worse performance or required low cutoff.

Subsequently, we also tested combinations of two and three fingerprints for target prediction, where the average of the component scores was taken as the ligand similarity score. As shown in Additional file 1: Figure S3, the combined fingerprints Morgan + MACCS (MM) and Morgan + MACCS + Daylight (MMD) performed better than MACCS and Daylight alone and combined Morgan + Daylight (MD) and MACCS + Daylight (MAD) with improved correct top 10 predictions. In addition, they achieved similar performances as Morgan, however, with increased optimal cutoff range (between 0.1 and 0.4). Consequently, we considered both combined fingerprints in further experiments and took the borderline cutoff of 0.4 as default in the LigTMap workflow.

### LigTMap target prediction performance evaluation

The prediction performance of the complete LigTMap workflow was assessed using the validation and benchmark sets. Because the binding IFP calculation is computationally expensive, we tested target classes from the validation set, of which benchmark data were also available. These included kinase, beta-secretase, BRD, CA, ligase, HIV, and TB. In total, 733 ligands were tested from the validation set and 98 from the benchmark set. The entire target prediction workflow was run comprising the MM or MMD for the ligand similarity search with IFP based binding similarity analysis. The predicted targets were ranked based on the LigTMap score, which was calculated as a weighted sum of the ligand and binding similarity scores. The results of different weights showed that the combined score from 70% of the ligand fingerprint score and 30% of the binding fingerprint score performed well in the identification of targets than using either only the ligand fingerprint or binding fingerprint alone. Furthermore, according to the SEA target prediction study, target class-specific models improve the prediction precision rate in ligand-based methods [37]. To verify this hypothesis, we compared the results from the all-target class prediction to those from the class-specific prediction (CS). In the CS experiment, each ligand was predicted for its target class only, and thus, the output contained only targets from this class. Table 2 shows the overall performance of LigTMap in the conducted experiments.

**Table 2:** Overall performance of LigTMap using MM or MMD as ligand similarity search, for all-target class prediction or class-specific (CS) prediction

	Average success rate			Average (Top 10)		
	Top 1	Top 5	Top 10	Precision	Recall	F1 score
Validation set						
MM	0.56	0.68	0.72	0.35	0.27	0.23
MMD	0.53	0.70	0.73	0.35	0.25	0.20
MM-CS	0.63	0.75	0.77	0.55	0.52	0.45
MMD-CS	0.61	0.74	0.76	0.54	0.38	0.35
Benchmark set						
MM	0.40	0.59	0.63	0.29	0.27	0.26
MMD	0.44	0.64	0.65	0.33	0.27	0.24
MM-CS	0.60	0.74	0.84	0.53	0.52	0.65
MMD-CS	0.57	0.73	0.82	0.55	0.45	0.44

For all-target class prediction, the LigTMap score with MMD achieved higher top-5 and top-10 success rates than that with MM in both validation and benchmark experiments. It achieved an average top-10 success rate of almost 70%, with an average precision rate of 0.34 and recall of 0.26. Notably, the comparison between all-target class experiments (LigTMap score with MM or MMD) and CS experiments (with MM-CS or MMD-CS) revealed significant improvement in all measures in the latter experiments. This is presumably due to the exclusion of “off-targets” from the prediction list. Moreover, the comparison between MM-CS and MMD-CS shows that the LigTMap score with MM-CS has a 1%–5% higher success rate and 15%–35% improvement in recall with comparable precision. Meanwhile, comparing to MMD, the LigTMap score with MM-CS improved the top-1 success rate by 27% and the top-10 success rate by 17%, with > 50% increase in precision. Overall, the LigTMap score with MMD outperformed that with MM in all-target class predictions; however, the LigTMap score with MM-CS surpassed MMD-CS in the CS predictions.

Table 3 demonstrates the detailed prediction performance of LigTMap for each target class. Among the seven target classes, in the validation set, the highest success rate was achieved for beta-secretase (> 0.9), followed by BRD, HIV (~ 0.8), CA and ligase (~ 0.7), kinase, and TB (~ 0.5). For the benchmark set, LigTMap performed exceptionally for CA, beta-secretase, and BRD (> 0.9). It also showed good performance for HIV (> 0.8) and moderate for kinase (~ 0.7). LigTMap failed to find correct targets for ligase and TB ligands. Consistent with Table 2, the LigTMap score with MMD performed better than MM in all-target class experiments for most targets in terms of the top 10 success rate, in expense of the reduced F1 score. As a target prediction method, successful identification of the query compounds is of great importance; thus, LigTMap score with MMD is suitable. For compounds the target class of which is known but not the proteins, MM-CS is more optimal for finding the correct target within the class. The prediction results of all benchmark compounds with their experimental targets, predicted targets, PDB IDs, and ranks of the first true targets are provided in Additional file 1: Tables S3–S9.

**Table 3:** Prediction performance for each target class in all-target class and class-specific experiments

	LigTMap score (MM)		LigTMap score (MMD)		LigTMap score (MM-CS)		LigTMap score (MMD-CS)	
	Top 10	F1 Score	Top 10	F1 Score	Top 10	F1 Score	Top 10	F1 Score
Validation set								
Kinase	0.52	0.20	0.54	0.16	0.55	0.32	0.56	0.19
Beta-secretase	0.97	0.29	0.97	0.23	1.00	0.54	1.00	0.42
Bromodomain	0.79	0.25	0.82	0.25	0.91	0.63	0.88	0.55
Carbonic anhydrase	0.74	0.19	0.72	0.23	0.74	0.29	0.72	0.28
Ligase	0.72	0.34	0.72	0.23	0.72	0.72	0.72	0.4
HIV	0.79	0.14	0.83	0.13	0.83	0.18	0.85	0.17
TB	0.49	0.53	0.21	0.20	0.62	0.58	0.44	0.42
Benchmark set								
Kinase	0.67	0.15	0.72	0.13	0.89	0.23	0.72	0.22
Beta-secretase	0.89	0.42	1.00	0.28	0.95	0.49	0.95	0.30
Bromodomain	0.89	0.27	0.95	0.33	0.95	0.72	0.95	0.70
Carbonic anhydrase	1.00	0.54	1.00	0.60	1.00	0.81	1.00	0.85
Ligase	0.00	0.00	0.00	0.00	0.20	0.00	0.20	0.00
HIV	0.80	0.29	0.80	0.31	1.00	0.83	1.00	0.93
TB	0.18	0.13	0.09	0.01	0.91	1.49	0.91	0.08

### Negative compounds prediction

To evaluate the performance of LigTMap on the prediction of negative compounds that resemble true ligands, we composed the non-native decoy sets from the DUD-E database [39] for the five benchmark targets: Beta secretase I, Bromodomain IV, HIV reverse transcriptase, Carbonic anhydrase II and Kinase PLK 1. Though selected randomly, these decoys were chosen based on their different molecular weights to ensure that the list is unbiased. Finally, 50 decoys were selected for each target and totally 250 decoys were run for LigTMap predictions. As shown in Table 4, LigTMap performed well in the non-native decoy prediction for the Carbonic anhydrase 2, HIV reverse transcriptase and Kinase PLK1, because only 2–6% of the non-native decoys were falsely identified with the intended target within the top ranking list. The results of Bromodomain and Beta secretase decoys are less satisfactory as 28% and 42% of the decoys (respectively) have predicted with the target. Nonetheless, these results are in sharp contrast to native ligand predictions, in which 72% to 97% of the ligands have their targets correctly predicted.

**Table 4:** LigTMap prediction on the 250 decoys for the 5 protein targets from the benchmark target classes

Intended target	Top 1	Top 5	Top 10	Fail	%Top-10	%Fail
Beta secretase I	2	9	14	36	28%	72%
Bromodomain IV	3	14	21	29	42%	58%
Carbonic anhydrase II	0	2	3	47	6%	94%
HIV Reverse transcriptase	0	1	1	49	2%	98%
Kinase PLK1	0	1	2	48	4%	96%

### Comparison to the state-of-the-art target prediction methods

**Fig. 2:**
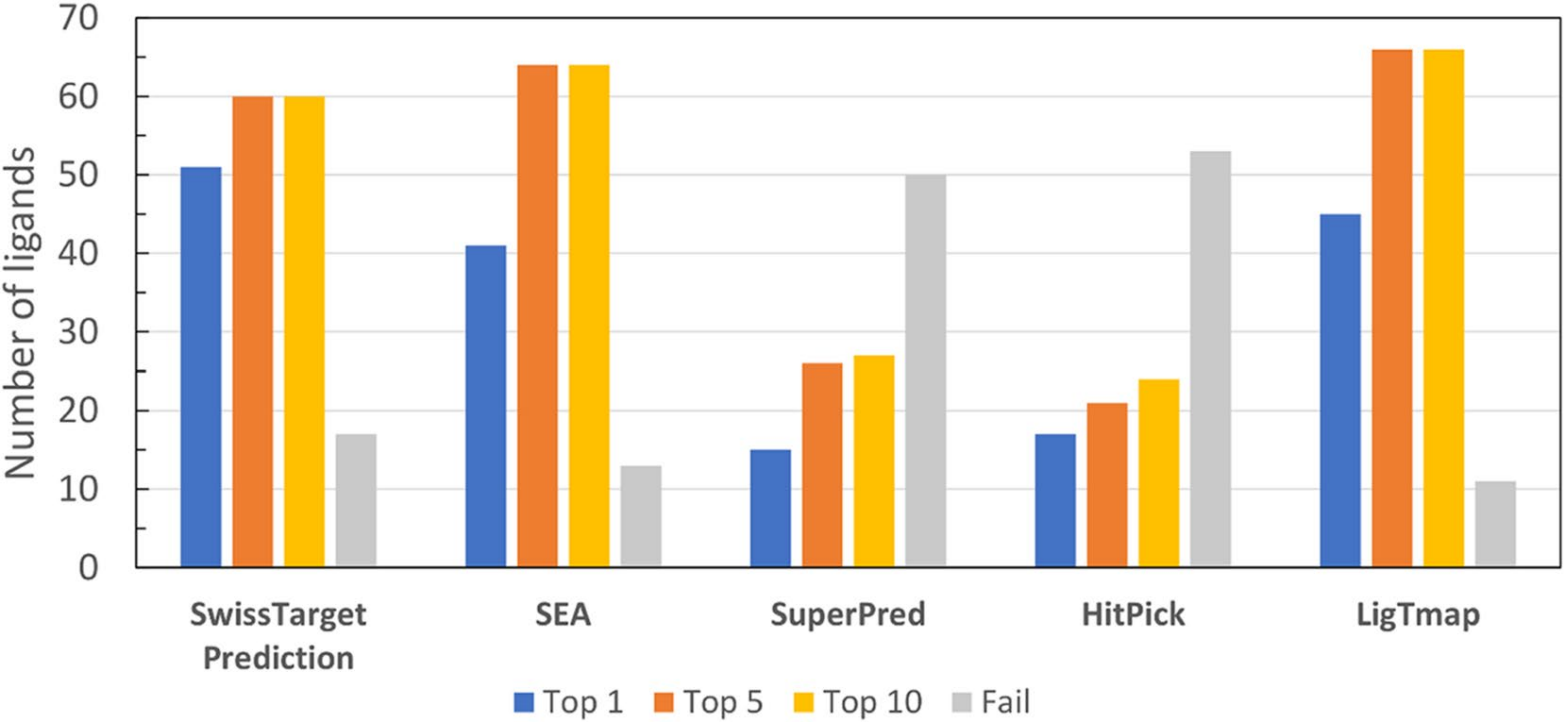
Comparative performance of five target prediction methods using benchmark compounds of human protein targets

The benchmark dataset was tested using four state-of-the-art target prediction servers, i.e., SwissTargetPrediction [19, 20, 22], SEA [11, 37], SuperPred [12], and HitPick [15]. As these servers mainly provide predictions for human targets, we excluded nonhuman targets from the comparative study. In total, 77 ligands for kinase, beta-secretase, CA, ligase, and BRD were evaluated. As the benchmark experiment was run against all classes, the LigTMap score with MMD was used and the results are shown in Fig. 2. LigTMap exhibited the highest top-10 success rates of 86%, followed by SEA (83%), and SwissTargetPrediction (78%). Moreover, it outperformed SuperPred and HitPick in all top-1, top-5, and top-10 success rates. SwissTargetPrediction has the highest top-1 success rate of 66%.

Regarding the predictions for each target class, as shown in Table 5, all beta-secretase ligands were successfully predicted by SwissTargetPrediction, SEA, and LigTMap. The same outcomes were noted for BRD, apart from LigTMap, which had one failure. For kinase, SEA successfully predicted all ligands; however, SwissTargetPrediction and LigTMap resulted in five failures. Furthermore, for CA target, LigTMap was the only method, which predicted all ligands, with the remaining four methods predicting just half of the cases. Ligase proved to be the most challenging target in the benchmarking experiment; no methods provided successful prediction for this target class. Based on the conducted analyses, it can be concluded that LigTMap reached the state-of-the-art performance and, in some target classes, outperformed the existing methods.

**Table 5:** The number of top-1, top-10, and failure predictions in the benchmark set for five target prediction servers

	SwissTargetPrediction			SEA			SuperPred			HitPick			LigTMap		
	Top 1	Top 10	Fail	Top 1	Top 10	Fail	Top 1	Top 10	Fail	Top 1	Top 10	Fail	Top 1	Top 10	Fail
Beta-secretase	16	19	0	17	19	0	9	19	0	6	6	13	15	19	0
Bromodomain	17	19	0	19	19	0	0	0	19	0	0	19	14	18	1
Kinase	10	13	5	1	18	0	0	0	18	10	10	8	3	13	5
Carbonic anhydrase	8	9	7	4	8	8	6	7	8	1	5	8	13	16	0
Ligase	0	0	5	0	0	5	0	0	5	0	0	5	0	0	5
Total	51	60	17	41	64	13	15	27	50	17	24	53	45	66	11
(%)	66	78	22	53	83	17	19	35	65	22	31	69	59	86	14

### Performance of Ligand Binding Activity Prediction

The prediction performance of all-target class-specific RF models using the core set is presented in Table 6. Two metrics were used to measure the performance of the models, i.e., the Pearson’s correlation coefficient (R) and the RMSE between the experimentally measured binding constants and the predicted values. Both coefficients were obtained by averaging from the test folds in the outer CV loop. The nested CV run was performed 10 times for each target, and the average performance and standard deviation were reported. Based on the obtained outcomes, we observed that R ranges between 0.5 and 0.8 for different target classes, with the HIV model achieves the highest correlation of 0.81 and the estrogen model the lowest correlation of 0.47. Overall, the average performance gives a correlation of 0.61 and RMSE of 1.23 (−log M).

**Table 6:** Cross-validation performance of 17 target class-specific RF activity prediction models on the core dataset

Target Class	Pearson’s Correlation Coefficient	RMSE (− log M)
HIV	0.81 ± 0.01	1.28 ± 0.03
Beta-secretase	0.75 ± 0.01	0.97 ± 0.03
Ligase	0.73 ± 0.01	1.26 ± 0.03
TB	0.67 ± 0.02	1.24 ± 0.03
Transferase	0.67 ± 0.01	1.30 ± 0.02
Kinase	0.66 ± 0.06	1.18 ± 0.01
HCV	0.65 ± 0.02	1.22 ± 0.04
Bromodomain	0.65 ± 0.02	0.93 ± 0.02
Carbonic anhydrase	0.64 ± 0.02	1.23 ± 0.04
Anticoagulant	0.63 ± 0.02	1.37 ± 0.03
Hydrolase	0.62 ± 0.02	1.42 ± 0.03
*Helicobacter pylori*	0.60 ± 0.11	1.44 ± 0.19
Influenza	0.59 ± 0.05	1.77 ± 0.08
Diabetes	0.53 ± 0.03	1.05 ± 0.03
Isomerase	0.53 ± 0.03	1.38 ± 0.01
Peroxisome	0.48 ± 0.07	0.99 ± 0.10
Estrogen	0.47 ± 0.07	1.12 ± 0.05
Average	0.61	1.23

The class-specific RF models were further assessed using the benchmark set. As shown in Table 7, the average correlation is 0.63, while RMSE is 1.26, which is in good agreement with the CV result. The two cases that are found different between the benchmark and validation results are the beta-secretase (benchmark/validation 0.31/0.75) and kinase (0.18/0.66). For the beta-secretase, the experimental values of the test compound concentrated in a narrow range of 7.6–8.2, whereas the prediction gave a range of 7.2–9. Despite the poor correlation, the RMSE for beta-secretase is low (only 0.49), suggesting that the predicted values were reasonably close to the experimental ones. Among the benchmark target classes, the kinase class is the most challenging one because it is a class with many diverse proteins. The experimental and prediction activities have only a correlation of 0.18 and RMSE of 1.62. A similar problem was reported in the SEA’s study [11]. It is reported that to improve the results with the kinase model, subclass specific models should be built. However, in our case, as we do not have enough data from PDBbind for each subclass of kinase, therefore the current poor performance of LigTMap for kinase cannot be improved until new data is available.

**Table 7:** Test performance of 7 target class-specific RF activity prediction models on the benchmark dataset

Target class	Pearson’s correlation coefficient	RMSE (− log M)
Bromodomain	0.95	0.65
Ligase	0.88	0.99
HIV	0.83	0.44
Carbonic anhydrase	0.57	1.92
TB	0.72	2.71
Beta-secretase	0.31	0.49
Kinase	0.18	1.62
Average	0.63	1.26

In the kinase class, some of the compounds were identified experimentally to target two different kinase proteins (PLK1 and ALK), and some also targeted another class, i.e., bromodomain BRD4. Taking the average of the PLK1 and ALK activity values and comparing them to the predicted values gave a poor correlation of 0.18 and RMSD of 1.62. Nevertheless, the predictions correlated better with PLK1 alone, giving an improved correlation of 0.61 and RMSD of 0.85 (see Additional file 1: Table S14). However, worse outcomes were noted when the predictions were correlated with ALK alone (correlation of − 0.33 and RMSD of 1.11). As none of the dual-class compounds were identified for BRD, the predicted activity using the BRD model poorly correlated with the experimental data for BRD4 (correlation of − 0.09 and RMSD of 1.17).

In the case of TB, although all compounds were identified to target both the bacterial TB (MtbAdok) and human kinase (hAdoK), only the TB model correlated reasonably with the MtbAdok activity (correlation of 0.72 and RMSD of 2.71). The kinase model did not correlate with hAdoK (correlation of 0.24 and RMSD of 5.42).

The activity prediction results of the benchmark set are listed in Additional file 1: Tables S9–S15.

### Case study of the HIV drug target prediction

Structure-based drug design has played an important role in the anti-HIV drug search. Since the first discovery of HIV 36 years ago [40] more than 30 drugs for the treatment of AIDS have been developed and identified to target all 7 life cycles of the virus [41]. The fast development of novel anti-HIV agents targeting multiple targets can be attributed to the availability of the 3D structures of the HIV proteins. In the PDBbind dataset alone, there are currently 580 crystal complexes of the HIV proteins with ligands. The structural information, together with accurate experimental activity data of true binders, can be exploited to construct highly accurate predictive HIV models to support the hits discovery and lead optimization. In the present study, the developed predictive RF model for HIV yields an average correlation of 0.81 with RMSE of 1.28 (− log M) in the nested CV assessment (see Table 5). Further evaluation of the RF model was performed using 10 novel compounds reported by Pribut et al. [42]. According to their study, aryl substituted benzimidazolones were experimentally validated to exhibit inhibitory effects against the HIV-1 non-nucleoside reverse transcriptase, with the reported pIC50 values in the range of 4.87–7.58. All 10 compounds have mean similarity scores range from 0.122 to 0.172 to our HIV core set (524 compounds) based on the Morgan fingerprint. The binding modes of these compounds were studied by molecular docking using the receptor structures PDB 2jle and 2fr2. Notably, our RF model for HIV displayed remarkable performance in predicting the activities of these 10 compounds, yielding a high correlation of 0.83 and RMSE of 0.44 (see Table 6).

**Fig. 3:**
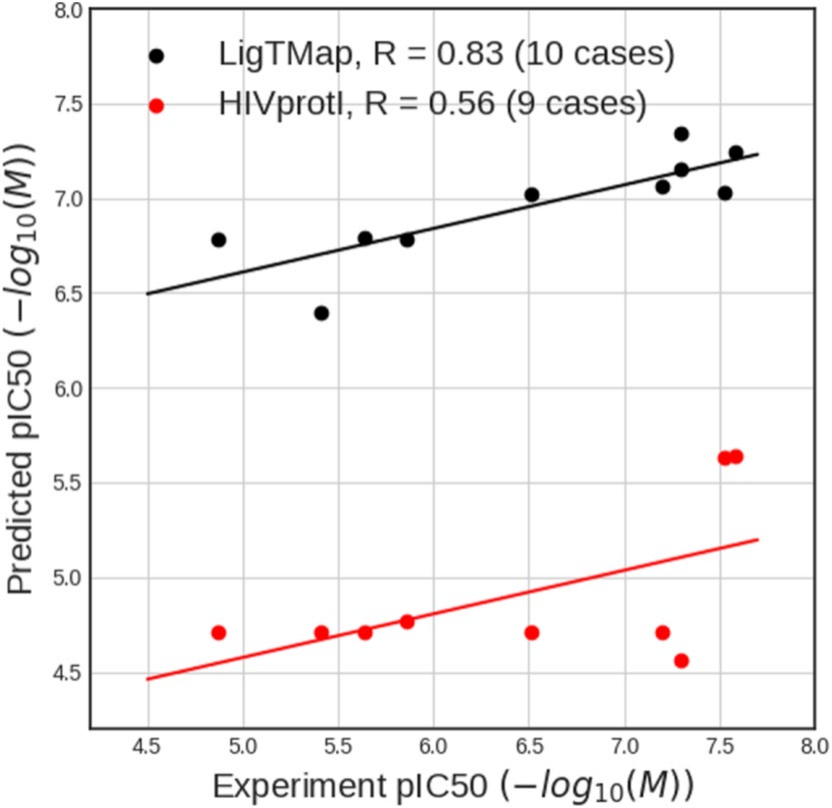
Performance comparison of HIV activity prediction methods

Furthermore, for comparison, we also tested the new compounds using two recently released online servers for anti-HIV biological activity prediction, namely, AntiHIV-Pred [43] and HIVprotI [44]. Their methods employed large-scale experimental data extracted from the ChEMBL database and their prediction models are ligand-based and HIV protein-specific. Users can select the prediction target as HIV protease, reverse transcriptase, integrase, REV (AntiHIV-Pred only), or TAT (AntiHIV-Pred only). Surprisingly, the AntiHIV-Pred server reported that the evaluated compounds were in the non-applicable domain, regardless of the selected target; therefore, no activity values were predicted. On the other hand, the HIVprotI server successfully returned prediction results for nine compounds against the reverse transcriptase target. However, poor correlation of 0.56 was obtained, which was significantly lower than the originally reported correlation of 0.76 [44]. Figure 3 showed a comparison of the anti-HIV inhibitor predictions by HIVprotI and LigTMap, indicating that LigTMap performed superior in activity predictions.

**Fig. 4:**
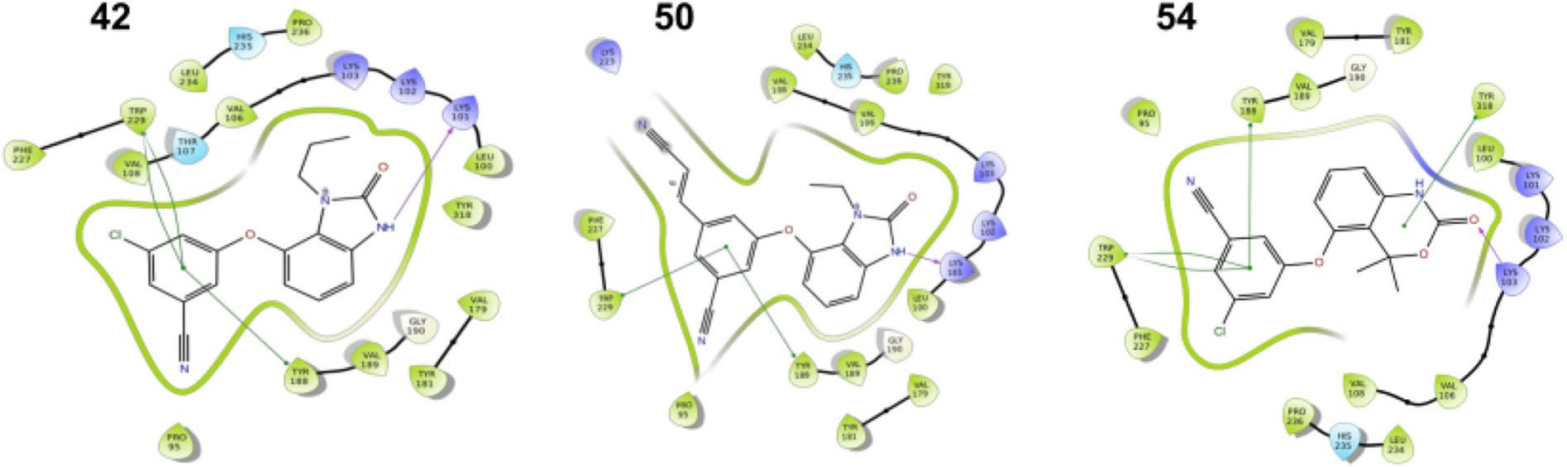
Predicted binding modes of three known anti-HIV ligands by PSOVina2 in LigTMap. Images were prepared using Schrödinger Maestro

We compared the binding modes predicted by PSOVina2 in LigTMap to the previously reported binding modes [42]. The three compounds were evaluated: **42**, **50**, and **54**. Previously, these compounds were lead optimized from the non-nucleoside reverse transcriptase inhibitor (PDB ID 2jle). Remarkably, LigTMap was able to predict the correct target for the three compounds in the top 1. As shown in Fig. 4, our predicted binding modes matched closely to the reported protein–ligand interaction patterns. For instance, the binding of the second-generation benzimidazole inhibitor (compound **42**) was reported to involve two pi-pi interactions with the Tyr188 and Trp229 residues, and a hydrogen bond to Lys101. The ligand docked by LigTMap retained an analogous binding mode, also predicting two pi-pi interactions and a hydrogen bond as the major contributors to the ligand binding. The reported binding modes of **50** involved the Lys101, Tyr188, Lys223, and Tyr229 amino acids, while Lys101, Tyr188, and Tyr229 participated in the interactions with compound **54**. Notably, all of them were correctly predicted by LigTMap.

### Features of the LigTMap server

The LigTMap server is free for non-commercial use at https://cbbio.online/LigTMap. The server accepts queries for multiple compound predictions (maximum of 20 for batch submissions) for both human and non-human (viral and bacterial) target classes. The output of the target prediction displays the name of the predicted target, its PDB ID, the ligand similarity score, binding similarity score, predicted activity value, docking score, and docking pose determined using PSOVina2. In Table 8, the LigTMap server is compared to the existing state-of-the-art target prediction servers with respect to their target scope and prediction output.

**Table 8:** Comparison of various features of target prediction servers

Server feature	SEA	SwissTarget Prediction	SuperPred	HitPick	LigTMap
Target scope					
Predict human targets	Yes	Yes	Yes	Yes	Yes
Predict non-human (viral and bacterial) targets	No	Yes (few)	No	No	Yes
Prediction					
Support input of multiple compounds	No	No	No	Yes	Yes
Target name	Yes	Yes	Yes	Yes	Yes
Target PDB	No	No	Yes	No	Yes
Biological activity	No	No	No	No	Yes
Binding mode	No	No	No	No	Yes
External links to target related information	ZINC	Uniprot, GeneCard	Uniprot, BindingDB, RefSeq, etc	GeneCard	PDB

## Conclusion

Target prediction of small molecules is a crucial step in drug discovery and study of disease mechanisms. The existing computational approaches to target prediction are limited in terms of availability, functionality, and accuracy. In the current work, we present LigTMap, a new target prediction method developed to predict 17 therapeutic protein classes, including human and nonhuman protein targets. It is a multistage prediction workflow, which combines the ligand similarity search with docking and binding similarity analysis to accurately identify protein targets. Extensive experiments utilizing validation and benchmark sets revealed that LigTMap (MMD) achieved a top-10 success rate of almost 70%, with an average precision rate of 0.34. This performance is good as compared to the current best prediction servers SwissTargetPrediction and SEA. Class-specific target prediction of LigTMap (MM-CS) improved the top-1 success rate by 27% and the top-10 success rate by 17%, with > 50% increase in precision. Hence, LigTMap is a new and reliable method for target prediction of novel ligands. Furthermore, it can identify with a higher success rate for ligands whose target class is known but the actual targets are still unknown.

The current version of LigTMap is limited to target classes from PDBbind. For future work, other large databases, such as ChEMBL and ZINC, as well as protein–ligand interaction databases, e.g., STITCH, will be exploited to expand the target class coverage and enhance the prediction accuracy.

## Supplementary Information

**Additional file 1.** Method description, benchmark data and prediction results.

## Data Availability

The LigTMap web server is free to use at https://cbbio.online/LigTMap. The source code is released on GitHub (https://github.com/ShirleyWISiu/LigTMap) under the BSD 3-Clause License to encourage re-use and further developments. The benchmark dataset is listed in additional file.
