# Supplementary material for: LigTMap: ligand and structure-based target identification and activity prediction for small molecular compounds
Source: J Cheminform. 2021 Jun 10;13:44. doi: 10.1186/s13321-021-00523-1 (PMC8194164; doi:10.1186/s13321-021-00523-1)
Supplement: Supplementary file 1 — Additional file 1. Method description, benchmark data and prediction results. [file 13321_2021_523_MOESM1_ESM.docx]

**Supplementary Material**

**LigTMap: Ligand and structure-based target identification and activity prediction for small molecular compounds**

Faraz Shaikh^1^, Hio Kuan Tai^1^, Nirali Desai^2^, Shirley W. I. Siu^1^*

^1^Department of Computer and Information Science, Faculty of Science and Technology, University of Macau, Avenida da Universidade, Taipa, Macau, China

^2^Division of Biological and Life Sciences, Ahmedabad University, Ahmedaba, India

*Corresponding Author:

E-mail: shirleysiu@um.edu.mo

Table of Contents

**Section I. Existing Target Prediction Servers** Page

Table S1: List of existing target prediction servers 2-3

**Section II. Methods**

Training-and-optimization of binding activity prediction models 4-6

Figure S1: The nested CV process to estimate the predictive error of the training-and-optimization procedure 4

**Section III. Results**

Figure S2-S3: Comparison of fingerprint algorithms for ligand similarity search 7-8

Table S2-S8 Target prediction results for the benchmark sets 9-15

Table S9-S15: Activity prediction results for the benchmark sets 16-23

**References**

**Supplementary Section I. Existing Target Prediction Servers**

**Table S1. List of existing target prediction servers**

**(A) Categorization of the servers by their methodology**

| **Method** | **Approach** | **Methodology** | **Dataset**  **No. of Target if Known** | **Online Server** | **Year** |
| --- | --- | --- | --- | --- | --- |
| Zhang | Structure-based | *Selection*: PharmMapper/IVS2vec/ LiSiCA  *Docking*: AutoDockVina  *Ranking*: Vina score | - | ✖ | 2020 |
| ACID | Structure-based | *Docking*: AutoDockVina + LEPHAR + PLANTS + PSOVina  *Ranking*: X-score and MM/PBSA | PDBbind core-set  809 | ✔ | 2019 |
| PatchSearch | Structure-based | *Selection*: Binding site similarity  *Docking*: smina  *Ranking*: Vinardo | PDB  - | ✔ | 2019 |
| TarPred | Ligand-based | *Fingerprint:* ECfp4  *Similarity:* Tc with data fusion | Binding DB  533 | ✖ | 2015 |
| SPiDER | Ligand-based + SOM | *Fingerprint:* pharmacophore, physiochemical properties  *Similarity:* Euclidean distance, P value | COBRA  - | ✖ | 2014 |
| SwissTarget  Prediction | Ligand based + Machine learning | *Fingerprint:* FP2, ES5D  *Similarity:* Logistic regression combined score | ChEMBL 23  >3000 | ✔ | 2013 |
| Target Hunter | Ligand-based + Machine learning | *Fingerprint:* ECfp6, ECfp4, FP2  *Similarity:* Tc, Logistic regression probability | ChEMBL 11  794 | ✔ | 2013 |
| HitPick | Ligand-based + Machine learning | *Fingerprint:* FCFP  *Similarity:* Tc, 1NN similarity search and Laplacian-modified naïve Bayesian | STITCH 3.1  1,375 | ✔ | 2013 |
| ChemMapper | Ligand and Structure-based | *Fingerprint:* FP2, MACCS, pharmacophore  *Similarity:* SHAFTS, USR | ChEMBL 14, BindingDB, Drug Bank, KEGG and PDB  >20,000 | ✔ | 2013 |
| DRAR-CPI | Structure-based | *Docking*: DOCK  *Ranking*: 2DIZ | UniProt, Drug Bank  385 | ✔ | 2011 |
| ChemProt | Ligand-based + Machine learning | *Fingerprint*: Daylight-like fps  *Similarity*: Naives Bayes QSAR ensemble | ChEMBL 19, Binding DB, PDSP Ki, DrugBank, PharmGKB, IUPHAR-DB, STITCH ATC, Slider 2  >20,000 | ✔ | 2010 |
| PharmMapper | Structure-based | *Fingerprint:* Receptor pharmacophore  *Similarity:* Fit score | TargetBank, DrugBank, BindingDB, PDTD  23,236 | ✔ | 2010 |
| PASS | Ligand-based | *Fingerprint*: Multilevel neighborhoods of atoms (MNA)  *Similarity:* Statistical | WDI, ACD  3,678 | ✔ | 2000 |
| SuperPred | Ligand-based | *Fingerprint*: ECFP4  *Similarity*: Tc, Z-score, E-value | SuperTarget, ChEMBL, Binding DB  1,800 | ✔ | 2008 |
| SEA | Ligand-based | *Fingerprint*: Daylight  *Similarity:* E-value, Max Tc | MDDR 2006.1  246 | ✔ | 2007 |
| TarFisDock | Structure-based | *Docking:* DOCK  *Ranking:* Interaction energy | PDTD  698 | ✖ | 2006 |

**(B) URLs of accessible (as of Feb 22, 2020) target prediction servers (in alphabetical order)**

| **Server Name** | **URL** |
| --- | --- |
| ACID | <http://chemyang.ccnu.edu.cn/ccb/server/ACID/> |
| ChemMapper | <http://lilab-ecust.cn/chemmapper/> |
| ChemProt | <http://potentia.cbs.dtu.dk/ChemProt/> |
| DRAR-CPI | <https://cpi.bio-x.cn/drar/> |
| HitPick | <http://mips.helmholtz-muenchen.de/hitpick/cgi-bin/index.cgi?content=hitIdentification.html> |
| PharmMapper | <http://www.lilab-ecust.cn/pharmmapper/> |
| PatchSearch | <https://bioserv.rpbs.univ-paris-diderot.fr/services/PatchSearch/> |
| PASS | <http://www.pharmaexpert.ru/passonline/index.php> |
| SEA | <http://sea.bkslab.org/> |
| SwissTargetPrediction | <http://www.swisstargetprediction.ch/> |
| SuperPred | <http://prediction.charite.de/> |
| TargetHunter | <https://www.cbligand.org/TargetHunter/search_target.php> |

**Supplementary Section II. Materials and Methods**

**Training-and-Optimization of Binding Activity Prediction Models**

Cross-validation (CV) is the most common approach used to estimate the generalization performance of ML models. Nonetheless, because the core datasets are typically small in size (in the range of ~50 to ~500, with the exception of kinase that has ~2000, to simultaneously tune the hyperparameters of the model and to provide a reliable estimate of the generalization performance, the nested CV procedure is more appropriate in this case (Stone, 1976). The purpose of the nested CV is to separate the data for testing from the data for training and validation to eliminate the issue of the information leak (Wainer and Cawley, 2018).


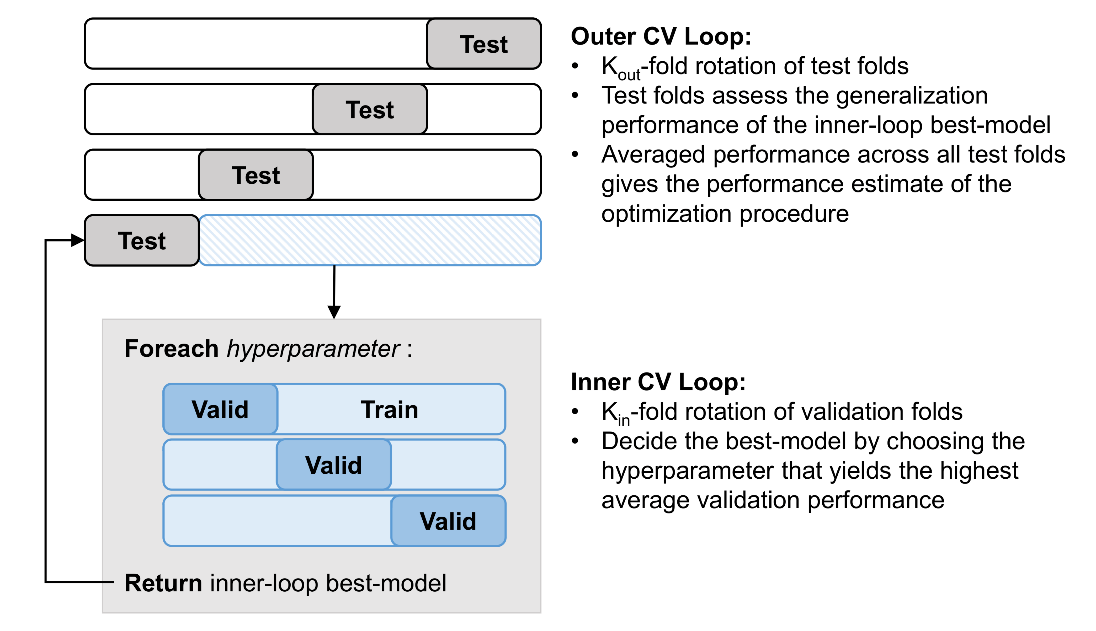


**Figure S1. The nested CV process to estimate the predictive error of the training-and-optimization procedure**

As demonstrated in Figure S1, it uses two CV loops to assess the parameter optimization procedure. In the inner CV loop, a model is constructed from the training fold, the prediction error of which is computed on the validation fold. A set of hyperparameters is given in turn to execute the inner CV loop, and the hyperparameter, which generates a model with the lowest error, is chosen as the most optimal model. Upon completion of the inner CV loop, the prediction performance of the best model is assessed in the outer CV loop using the left-out test fold. All test folds are rotated for the assessment of the generalization error of the best models of the inner loop. Finally, the averaged performance across all test folds affords the error estimate of the parameter optimization procedure, while the standard deviation assesses the variance of the procedure.

It is imperative to optimize two hyperparameters in a random forest regressor (RandomForestRegressor), i.e., the number of trees in the forest (n_estimators) and the depth of each tree in the forest (max_depth). Considering the former, the more trees in the forest, the better they can learn the data. The model variance often decreases with the increasing number of trees, and the prediction accuracy converges. However, numerous examples have demonstrated that more optimal models with fewer trees do exist, as there is a possibility to overfit with an unnecessarily large number of trees. Regarding the depth of the tree, the deeper the tree is, the more splits it has; therefore, it can capture more information about the data. Nevertheless, the deeper it is, the chance of the model being overfitted increases. As the target datasets are considerably smaller than many other ML approaches, the qualities of the trained models are highly sensitive to the chosen hyperparameters. Using the nested CV routine with a grid search allows for an unbiased estimate of the performance of the model, the hyperparameters of which are selected based on a CV procedure.

The ML model construction and CV experiments were implemented in Python 3.2 using scikit-learn v. 0.19. The KFold splitter from the sklearn library was utilized to generate CV folds with Kin = Kout = 4. The production model was obtained by fitting the entire dataset with hyperparameters that gave the lowest root-mean-square error (RMSE) in the 10-fold CV of the dataset (Stone, 1976)

**Supplementary Section III. Results**

**
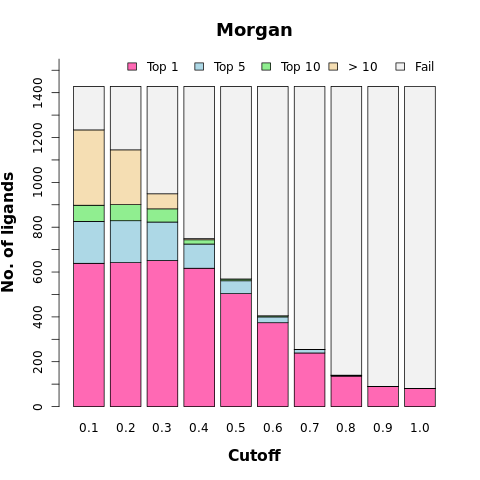

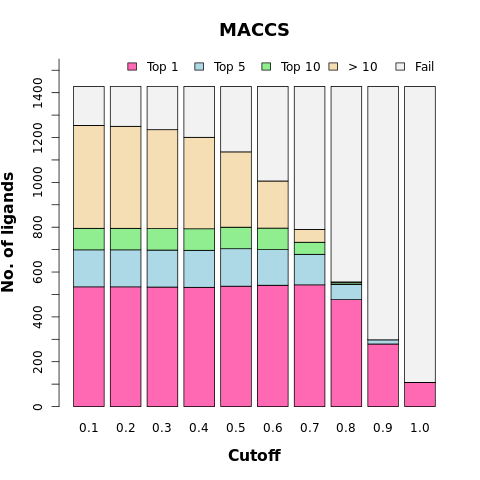
**

**
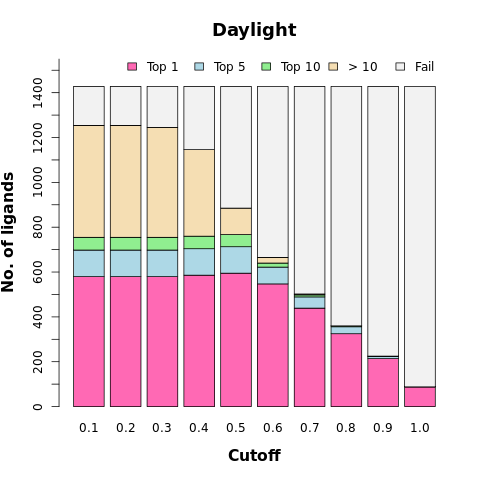

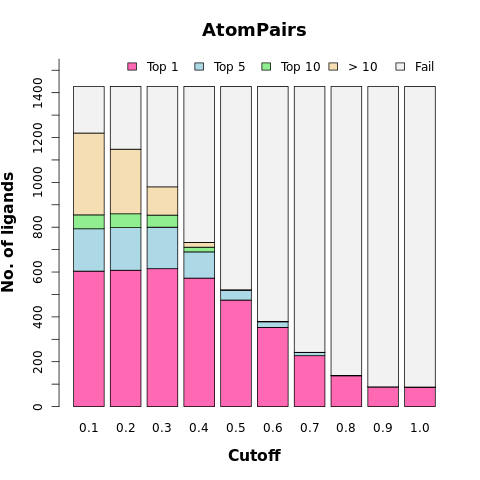
**

**
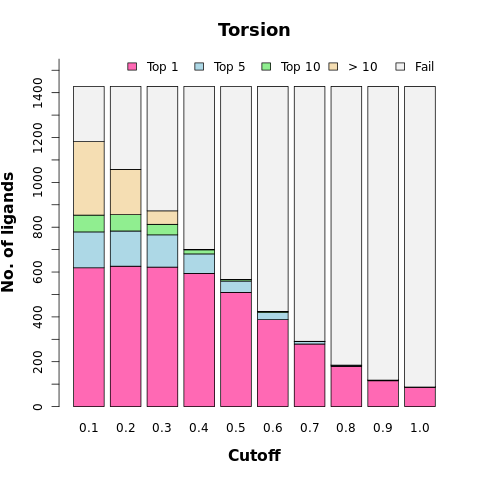

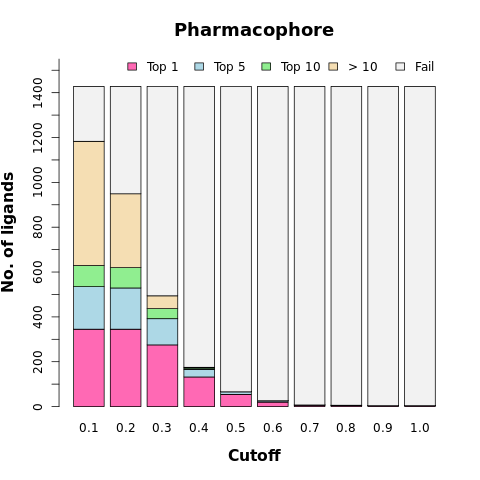
**

**Figure S2.** **Comparison of fingerprint algorithms for** **ligand similarity search**

**
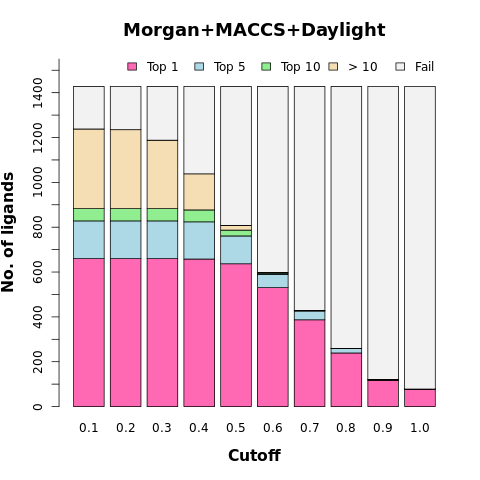

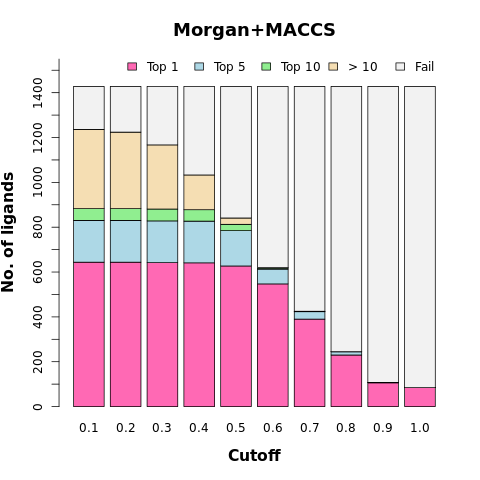
**

**
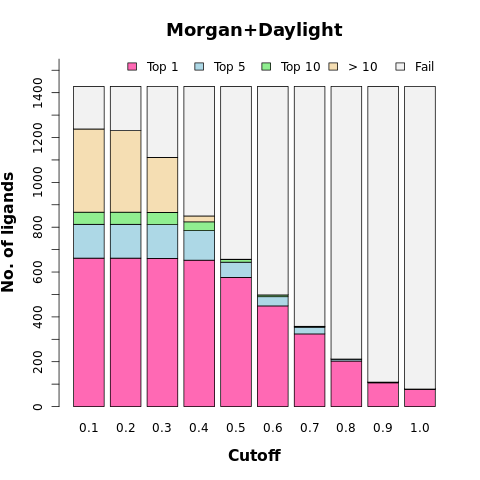

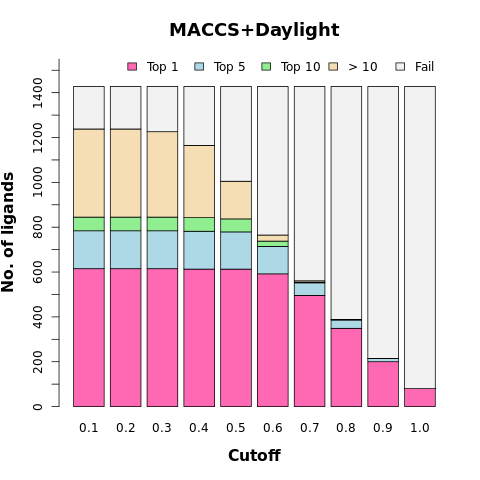
**

**Figure S3. Comparison of combined fingerprint algorithms for ligand similarity search**

**Table S2. Target Prediction for the Benchmark Data – Kinase**

Note: Some compounds were experimentally identified to have more than one target; ALK and PLK belong to the Kinase class and BRD4 belongs to the Bromodomain class

Source of experimental data: (Watts *et al.*, 2019)

| **Compound** | **SMILES** | **Expt target** | **Predicted target** | **Predicted PDB ID** | **Rank of prediction** |
| --- | --- | --- | --- | --- | --- |
| 16a | O=C1[C@@H](CC)N(C2CCCC2)C3=C(N1C)C=NC(NC4=C(OC)C=C(C5CCN(C)CC5)C=C4)=N3 | ALK, BRD4, PLK1 | PLK1 | 2rku | 3^rd^ |
| 16b | O=C1[C@@H](CC)N(C2CCCC2)C3=C(N1C)C=NC(NC4=C(OCC(C)C)C=C(C(NC5CCN(C)CC5)=O)C=C4)=N3 | ALK, BRD4 | PLK1 | 2owb | 61^st^ |
| 16c | O=C1[C@@H](CC)N(C2CCCC2)C3=C(N1C)C=NC(NC4=C(OCC)C=C(C(NC5CCN(C)CC5)=O)C=C4)=N3 | ALK, BRD4, PLK1 | PLK1 | 2rku | 1^st^ |
| 16d | O=C1[C@H](CC)N(C2CCCC2)C3=C(N1C)C=NC(NC4=C(OCC)C=C(C(NC5CCN(C)CC5)=O)C=C4)=N3 | ALK | PLK1 | 3fc2 | 3^rd^ |
| 16e | O=C1CN(C2CCCC2)C3=C(N1C)C=NC(NC4=C(OCC)C=C(C(NC5CCN(C)CC5)=O)C=C4)=N3 | ALK | PLK1 | 3fc2 | 3^rd^ |
| 16f | O=C1[C@@H](CC)N(CC2=CC(Br)=CC=C2)C3=C(N1C)C=NC(NC4=C(OCC)C=C(C(NC5CCN(C)CC5)=O)C=C4)=N3 | ALK, BRD4, PLK1 | PLK1 | 2rku | 1^st^ |
| 16g | O=C1[C@@H](CC)N(CC2=CC=CC=C2)C3=C(N1C)C=NC(NC4=C(OCC)C=C(C(NC5CCN(C)CC5)=O)C=C4)=N3 | ALK, BRD4, PLK1 | PLK1 | 2rku | 1^st^ |
| 16h | O=C1[C@@H](CC)N(CC2=CC(Br)=CC=C2)C3=C(N1C)C=NC(NC4=C(OCC)C=C(C5CCN(C)CC5)C=C4)=N3 | ALK, BRD4, PLK1 | ALK1 | 4fnz | 12^th^ |
| 16i | O=C1[C@@H](CC)N(CC2=CC=CC=C2)C3=C(N1C)C=NC(NC4=C(OCC)C=C(C5CCN(C)CC5)C=C4)=N3 | ALK, BRD4, PLK1 | PLK1 | 2rku | 2^nd^ |
| 23a | O=C1[C@@H](CC)N(CC2=CC(Cl)=CC=C2)C3=C(N1C)C=NC(NC4=C(OCC)C=C(C5CCN(C)CC5)C=C4)=N3 | ALK, BRD4, PLK1 | PLK1 | 2rku | 3^rd^ |
| 23b | O=C1[C@@H](CC)N(CC2=CC(C#N)=CC=C2)C3=C(N1C)C=NC(NC4=C(OCC)C=C(C5CCN(C)CC5)C=C4)=N3 | ALK, BRD4, PLK1 | PLK1 | 4j53 | 2^nd^ |
| 23c | O=C1[C@@H](CC)N(CC2=CC(OC)=CC=C2)C3=C(N1C)C=NC(NC4=C(OCC)C=C(C5CCN(C)CC5)C=C4)=N3 | ALK | PLK1 | 2owb | 146^th^ |
| 23d | O=C1[C@@H](CC)N(CC2=C(Br)C=CC=C2)C3=C(N1C)C=NC(NC4=C(OCC)C=C(C5CCN(C)CC5)C=C4)=N3 | ALK | PLK1 | 2rku | 3^rd^ |
| 23e | O=C1[C@@H](CC)N(CC2=CC=C(Br)C=C2)C3=C(N1C)C=NC(NC4=C(OCC)C=C(C5CCN(C)CC5)C=C4)=N3 | ALK | PLK1 | 4j53 | 3^rd^ |
| 16j | O=C1[C@@H](CC)N(CC2=C(C)C=CS2)C3=C(N1C)C=NC(NC4=C(OCC)C=C(C5CCN(C)CC5)C=C4)=N3 | ALK, BRD4, PLK1 | PLK1 | 4a4o | 22^nd^ |
| 16k | O=C1[C@@H](CC)N(CC2=CC(C)=CS2)C3=C(N1C)C=NC(NC4=C(OCC)C=C(C5CCN(C)CC5)C=C4)=N3 | ALK, BRD4, PLK1 | PLK1 | 2rku | 4^th^ |
| 16l | O=C1[C@@H](CC)N(CC2=CC=C(C)S2)C3=C(N1C)C=NC(NC4=C(OCC)C=C(C5CCN(C)CC5)C=C4)=N3 | ALK | PLK1 | 2rku | 4^th^ |
| 23f | O=C1[C@@H](CC)N(CC2=C(C)N=CS2)C3=C(N1C)C=NC(NC4=C(OCC)C=C(C5CCN(C)CC5)C=C4)=N3 | ALK | PLK1 | 2owb | 117^th^ |

**Table S3. Target Prediction for the Benchmark Data – Tuberculosis**

Note: Some compounds were experimentally identified to target more than one target class; MtbAdok belongs to the (bacteria) TB class and hAdok belongs to the (human) Kinase class

Source of experimental data: (Laura A T Cleghorn *et al.*, 2018)

| **Compound** | **SMILES** | **Expt target** | **Predicted target** | **Predicted PDB ID** | **Rank of prediction** |
| --- | --- | --- | --- | --- | --- |
| 1 | OC[C@@H]1[C@@H](O)[C@@H](O)[C@H](N2C=NC3=C2N=CC=C3N4CCN(C5=CC=C(C6=CC=CC=C6)C=C5)CC4)O1 | MtbAdok, hAdok | hAdok | 4o1l | 18^th^ |
| 2 | OC[C@@H]1[C@@H](O)[C@@H](O)[C@H](N2C=NC3=C2N=CN=C3N4CCN(C5=CC=C(C6=CC=C(OC)N=C6)C=C5)CC4)O1 | MtbAdok, hAdok | MtbAdok | 4pvv | 44^th^ |
| 3 | OC[C@@H]1[C@@H](O)[C@@H](O)[C@H](N2C=NC3=C2N=CN=C3N4CCN(C5=CC=C(C6=CC=C(C(C)(O)C)C=C6)C=C5)CC4)O1 | MtbAdok, hAdok | MtbAdok | 4pvv | 136^th^ |
| 4 | OC[C@@H]1[C@@H](O)[C@@H](O)[C@H](N2C=NC3=C2N=CN=C3C4=CC=C(C#CC5=CC=CC=C5)C=C4)O1 | MtbAdok, hAdok | hAdok | 4o1l | 24^th^ |
| 5 | OC[C@@H]1[C@@H](O)[C@@H](O)[C@H](N2C=NC3=C2N=CN=C3N4CCN(C5=CC=C(C6=CC=C(C(F)(F)F)C=C6)C=C5)CC4)O1 | MtbAdok, hAdok | hAdok | 4o1l | 13^th^ |
| 6 | OC[C@@H]1[C@@H](O)[C@@H](O)[C@H](N2C=NC3=C2N=CN=C3N4CCN(C5=CC=C(C6=CN(C(F)F)N=C6)C=C5)CC4)O1 | MtbAdok, hAdok | hAdok | 4o1l | 10^th^ |
| 7 | OC[C@@H]1[C@@H](O)[C@@H](O)[C@H](N2C=NC3=C2N=CN=C3N4CCN(C5=CC=C(C6=CC=CC(CN7CCOCC7)=C6)C=C5)CC4)O1 | MtbAdok, hAdok | MtbAdok | 4pvv | 5^th^ |
| 8 | OC[C@@H]1[C@@H](O)[C@@H](O)[C@H](N2C=NC3=C2N=CN=C3N4CCN(C5=CC=C(C6=CC=CC=C6)C=C5)CC4)C1 | MtbAdok, hAdok | hAdok | 4o1l | 28^th^ |
| 9 | OC[C@@H]1[C@@H](O)[C@@H](O)[C@H](N2C=NC3=C2N=CN=C3N4CCN(C5=CC=C(C6=CC=C(C(F)(F)F)N=C6)C=C5)CC4)O1 | MtbAdok, hAdok | hAdok | 2i6a | 99^th^ |
| 10 | OC[C@@H]1[C@@H](O)[C@@H](O)[C@H](N2C=NC3=C2N=CN=C3C#CC4=CC=C(C5=CC=CC=C5)C=C4)O1 | MtbAdok, hAdok | hAdok | 4o1l | 19^th^ |
| 11 | OC[C@@H]1[C@@H](O)[C@@H](O)[C@H](N2C=NC3=C2N=CN=C3N4CCN(C5=CC=C(C6=CC=CC=C6)C=C5)CC4)O1 | MtbAdok, hAdok | hAdok | 4o1l | 35^th^ |

**Table S4. Target Prediction for the Benchmark Data – Ligase**

Source of experimental data: (Jessica E Watt *et al.*, 2018)

| **Compound** | **SMILES** | **Expt target** | **Predicted target** | **Predicted PDB ID** | **Rank of prediction** |
| --- | --- | --- | --- | --- | --- |
| 1 | Oc1cc(C)c(O)cc1-c2c(O)cc(C)c(c2)O | WWP2 E3 Ubiquitin Ligase | Fail | Fail | Fail |
| 7 | [O-][n+]1ccccc1Sc2ccc([N+](=O)[O-])c(c23)non3 | WWP2 E3 Ubiquitin Ligase | Fail | Fail | Fail |
| 12 | O=C1C=CC(=O)N1C2=CC(=C[C@@H](C2)C(=O)O)N3C(=O)C=CC3=O | WWP2 E3 Ubiquitin Ligase | Fail | Fail | Fail |
| 19 | O=[N+]([O-])c1ccc(c(c12)non2)[S@](=O)c3ccc(C)cc3 | WWP2 E3 Ubiquitin Ligase | Fail | Fail | Fail |
| 20 | c1cccc(c12)n(CCOC)c3c(n2)c(=O)n(c(n3)=O)-c4ccccc4 | WWP2 E3 Ubiquitin Ligase | WWP2 E3 Ubiquitin Ligase | 5hmk | 30^th^ |

**Table S5. Target Prediction for the Benchmark Data – Bromodomain**

Source of experimental data: (Bamborough *et al.*, 2018)

| **Compound** | **SMILES** | **Expt Target** | **Predicted Target** | **Predicted PDB ID** | **Rank of Prediction** |
| --- | --- | --- | --- | --- | --- |
| **1** | Cc1cc2ccnc(N[C@H]3CCNC[C@@H]3OCC3CCCCC3)c2[nH]c1=O | ATAD2, BRD4 | BRD4 | 5lj2 | 1^st^ |
| **2** | Cc1cc2c(cnc(N[C@@H]3CCNC[C@H]3OCC3CCCCC3)c2[nH]c1=O)-c1cncc(N)c1 | ATAD2, BRD4 | BRD4 | 5lj1 | **1^st^** |
| **3** | Cc1cncc(c1)-c1cnc(N[C@@H]2CCNC[C@H]2OCC2CCCCC2)c2[nH]c(=O)c(C)cc12 | ATAD2, BRD4 | Bromodomain testis specific protein | 5lj1 | **1^st^** |
| **4** | Cc1cncc(c1)-c1cnc(N[C@@H]2CCNC[C@H]2OCC2CCS(=O)(=O)CC2)c2[nH]c(=O)c(C)cc12 | ATAD2, BRD4 | Bromodomain contatining G couple protein | 5a5s | **1^st^** |
| **5** | CO[C@H]1CNC[C@@H](OCC2CCS(=O)(=O)CC2)[C@@H]1Nc1ncc(-c2cncc(C)c2)c2cc(C)c(=O)[nH]c12 | ATAD2, BRD4 | BRD4 | 5lj1 | **1^st^** |
| **6** | CO[C@H]1CNC[C@@H](OCC2CCC(F)(F)CC2)[C@@H]1Nc1ncc(-c2cncc(C)c2)c2cc(C)c(=O)[nH]c12 | ATAD2, BRD4 | BRD4 | 5lj1 | **1^st^** |
| **8** | CN1CCC(CC1)Nc1nccc2cc(C)c(=O)[nH]c12 | ATAD2, BRD4 | BRD4 | 5a5s | **1^st^** |
| **9** | CN1[C@H]2CC[C@@H]1C[C@@H](C2)Nc1nccc2cc(C)c(=O)[nH]c12 | ATAD2, BRD4 | BRD4 | 5a5s | **11^th^** |
| **10** | CN1[C@H]2CC[C@@H]1C[C@H](C2)Nc1nccc2cc(C)c(=O)[nH]c12 | ATAD2, BRD4 | BRD4 | 5a85 | **2^nd^** |
| **21** | Cc1cncc(c1)-c1cnc(N[C@@H]2CCNC[C@H]2OCC2CCOCC2)c2[nH]c(=O)c(C)cc12 | ATAD2, BRD4 | CREB binding bromodomain | 5lj1 | **2^nd^** |
| **22** | CO[C@H]1CNC[C@@H](OCC2CCOCC2)[C@@H]1Nc1ncc(-c2cncc(C)c2)c2cc(C)c(=O)[nH]c12 | ATAD2, BRD4 | BRD4 | 5lj1 | **2^nd^** |
| **23** | Cc1cncc(c1)-c1cnc(N[C@@H]2C[C@@H]3CC[C@@H](N3)[C@H]2CCC2CCOCC2)c2[nH]c(=O)c(C)cc12 | ATAD2, BRD4 | BRD4 | 5a5s | **1^st^** |
| **24** | Cc1cncc(c1)-c1cnc(N[C@@H]2C[C@H]3CC[C@H](N3)[C@H]2CCC2CCS(=O)(=O)CC2)c2[nH]c(=O)c(C)cc12 | ATAD2, BRD4 | CREB binding bromodomain | 5lj1 | **1^st^** |
| **25** | Cc1cncc(c1)-c1cnc(N[C@@H]2CCNC[C@H]2OCC2CCC(F)(F)CC2)c2[nH]c(=O)c(C)cc12 | ATAD2, BRD4 | BRD4 | 5lj1 | **1^st^** |
| **26** | Cc1cncc(c1)-c1cnc(N[C@@H]2C[C@@H]3CC[C@@H](N3)[C@H]2CCC2CCC(F)(F)CC2)c2[nH]c(=O)c(C)cc12 | ATAD2, BRD4 | BRD4 | 5lj1 | **3^rd^** |
| **27** | Cc1cncc(c1)-c1ccc(N[C@@H]2C[C@@H]3CC[C@@H](N3)[C@H]2CCC2CCS(=O)(=O)CC2)c2[nH]c(=O)c(C)cc12 | ATAD2, BRD4 | BRD4 | 5a5s | **1^st^** |
| **28** | Cc1cncc(c1)-c1ccc(N[C@@H]2CCNC[C@H]2OCC2CCS(=O)(=O)CC2)c2[nH]c(=O)c(C)cc12 | ATAD2, BRD4 | BRD4 | 5lj1 | **1^st^** |
| **29** | CO[C@H]1CNCC[C@@H]1Nc1nccc2cc(C)c(=O)[nH]c12 | ATAD2, BRD4 | BRD4 | 5lj1 | **1^st^** |
| **30** | CO[C@@H]1CNC[C@H](OC)[C@H]1Nc1nccc2cc(C)c(=O)[nH]c12 | ATAD2, BRD4 | BRD4 | 5lj1 | **1st** |

**Table S6. Target Prediction for the Benchmark Data – Beta Secretase**

Source of experimental data: (Fujimoto *et al.*, 2019)

| **Compound** | **SMILES** | **Expt target** | **Predicted target** | **Predicted PDB ID** | **Rank of prediction** |
| --- | --- | --- | --- | --- | --- |
| 1 | C[C@]1(CS(N(C)C(N)=N1)(=O)=O)C2=CC(NC(C3=NC=C(F)C=C3)=O)=CC=C2F | Beta Secretase I | Beta Secretase I | 5hu1 | 1^st^ |
| 2 | NC1=N[C@]([C@]2([H])CS1)(CO[C@@H]2C)C3=CC(NC(C4=NC=C(C(F)F)N=C4)=O)=CC=C3F | Beta Secretase I | Beta Secretase I | 4x7i | 1^st^ |
| 3 | NC1=N[C@]2(C3=C(C[C@@]42CC[C@@H](OC)CC4)C=CC(C5=CN=CC(C#CC)=C5)=C3)N=C1C | Beta Secretase I | Beta Secretase I | 4b78 | 1^st^ |
| 4 | NC1=N[C@@](C2=CC(C3=CN=CN=C3)=CC=C2)(C4=CC(CC)=NC(CC)=C4)C(N1C)=O | Beta Secretase I | Beta Secretase I | 3in4 | 1^st^ |
| 8 | FC1=CC=C(NC(C2=NC=C(Cl)C=C2)=O)C=C1[C@]3(C)CCSC(N)=N3 | Beta Secretase I | Beta Secretase I | 5tol | 1^st^ |
| 9 | FC1=CC=C(NC(C2=NC=C(F)C=C2)=O)C=C1[C@]3(C)CCSC(N)=N3 | Beta Secretase I | Beta Secretase I | 5tol | 1^st^ |
| 10 | FC1=CC=C(NC(C2=NC=C(C#N)C=C2)=O)C=C1[C@]3(C)CCSC(N)=N3 | Beta Secretase I | Beta Secretase I | 4j0p | 1^st^ |
| 11 | FC1=CC=C(NC(C2=NC=C(C(F)F)N=C2)=O)C=C1[C@]3(C)CCSC(N)=N3 | Beta Secretase I | Beta Secretase I | 4j17 | 1^st^ |
| 12 | FC1=CC=C(NC(C2=NC=C(OC)N=C2)=O)C=C1[C@]3(C)CCSC(N)=N3 | Beta Secretase I | Beta Secretase I | 4j0p | 2^nd^ |
| 13 | FC1=CC=C(NC(C2=NC=C(OCC(F)(F)F)N=C2)=O)C=C1[C@]3(C)CCSC(N)=N3 | Beta Secretase I | Beta Secretase I | 4j0z | 2^nd^ |
| 14 | FC1=CC=C(NC(C2=NC=C(OCF)N=C2)=O)C=C1[C@]3(C)CCSC(N)=N3 | Beta Secretase I | Beta Secretase I | 4j17 | 1^st^ |
| 15 | FC1=CC=C(NC(C2=NC=C(OCF)N=C2)=O)C=C1[C@]3(C)[C@@H](OC)CSC(N)=N3 | Beta Secretase I | Beta Secretase I | 4j0v | 1^st^ |
| 16 | FC1=CC=C(NC(C2=NC=C(OCF)N=C2)=O)C=C1[C@]3(C)[C@@H](C4=CC=CC=C4)CSC(N)=N3 | Beta Secretase I | Beta Secretase I | 4j17 | 1^st^ |
| 17 | FC1=CC=C(NC(C2=NC=C(OCF)N=C2)=O)C=C1[C@]3(C)[C@H](C4=CC=CC=C4)CSC(N)=N3 | Beta Secretase I | Beta Secretase I | 3zmg | 1^st^ |
| 18 | FC1=CC=C(NC(C2=NC=C(OCF)N=C2)=O)C=C1[C@]3(C)[C@@H](C4CCOCC4)CSC(N)=N3 | Beta Secretase I | Beta Secretase I | 4j1e | 1^st^ |
| 19 | FC1=CC=C(NC(C2=NC=C(OCF)N=C2)=O)C=C1[C@]3(C)C4(CCOCC4)CSC(N)=N3 | Beta Secretase I | Beta Secretase I | 4jpe | 3^rd^ |
| 20 | FC1=CC=C(NC(C2=NC=C(OCF)N=C2)=O)C=C1[C@]3(C)C4(CC(F)(F)C4)CSC(N)=N3 | Beta Secretase I | Beta Secretase I | 4j0v | 2^nd^ |
| 21 | FC1=CC=C(NC(C2=NC=C(OCF)N=C2)=O)C=C1[C@]3(C)C4(CCC(F)(F)CC4)CSC(N)=N3 | Beta Secretase I | Beta Secretase I | 4r5n | 2^nd^ |
| 22 | FC1=CC=C(NC(C2=NC=C(OCF)N=C2)=O)C=C1[C@]3(C)C4(CCS(CC4)(=O)=O)CSC(N)=N3 | Beta Secretase I | Beta Secretase I | 4rro | 4^th^ |

**Table S7. Target Prediction for the Benchmark Data – Carbonic Anhydrase**

Source of experimental data: (Buemi *et al.*, 2019)(Andring *et al.*, 2019)

| **Class** | **SMILES** | **Expt Target** | **Predicted Target** | **Predicted PDB ID** | **Rank of Prediction** |
| --- | --- | --- | --- | --- | --- |
| 6H2Z/7a | O=S(=O)(N)c1ccc(cc1)C(=O)N(CC2)CCC2c3ccccc3 | Carbonic_Anhydrase II | Carbonic Anhydrase II | 2x7t | 1^st^ |
| 6H33/7b | O=S(=O)(N)c1ccc(cc1)C(=O)N(CC2)CCC2(O)c3ccccc3 | Carbonic_Anhydrase II | Carbonic Anhydrase II | 2gd8 | 1^st^ |
| 6H34/8b | O=S(=O)(N)c1ccc(cc1)C(=O)N2CCN(CC2)Cc3ccc(F)cc3 | Carbonic_Anhydrase II | Carbonic Anhydrase II | 2x7t | 1^st^ |
| STX49 | O=S(=O)(N)O[C@H]1CC[C@H]([C@@]12C)[C@H]3[C@H](CC2)c4c(CC3)cc(cc4)OS(=O)(=O)N | Carbonic Anhydrase II and IX | Carbonic Anhydrase II | 3bet | 1^st^ |
| STX140 | O=S(=O)(N)O[C@H]1CC[C@H]([C@@]12C)[C@H]3[C@H](CC2)c4c(CC3)cc(OS(=O)(=O)N)c(c4)OC | Carbonic Anhydrase II and IX | Carbonic Anhydrase II | 2x7s | 1^st^ |
| STX243 | O=S(=O)(N)O[C@H]1CC[C@H]([C@@]12C)[C@H]3[C@H](CC2)c4c(CC3)cc(OS(=O)(=O)N)c(c4)CC | Carbonic Anhydrase II and IX | Carbonic Anhydrase II | 3igp | 1^st^ |
| STX641 | N#CC[C@H]1CC[C@H]([C@@]12C)[C@H]3[C@H](CC2)c4c(CC3)cc(OS(=O)(=O)N)c(c4)OC | Carbonic Anhydrase II and IX | Fail | 4z1k | 3^rd^ |
| STX738 | O=S(=O)(N)O[C@H]1CC[C@H]([C@@]12C)[C@H]3[C@H](CC2)c4c(CC3)cc(O)c(c4)OC | Carbonic Anhydrase II and IX | Carbonic Anhydrase II | 4z1k | 1^st^ |
| STX2182 | COC1=C(OS(=O)(N)=O)C=C(CCN(C(C2=CC(OC)=C(OC)C=C2)=O)C3=O)C3=C1 | Carbonic Anhydrase II and IX | Carbonic Anhydrase II | 4z1k | 2^nd^ |
| STX2480 | OC1=C(OC)C=C2C(CCN(CC3=CC(OC)=C(OC)C(OC)=C3)C2)=C1 | Carbonic Anhydrase II and IX | Fail | 4z1n | 1^st^ |
| STX2484 | COC1=C(OS(=O)(N)=O)C=C(CCN(CC2=CC(OC)=C(OC)C(OC)=C2)C3)C3=C1 | Carbonic Anhydrase II and IX | Carbonic Anhydrase II | 2x7u | 1^st^ |
| STX2839 | OC1=CC=C2C(CCN(CC3=CC(OC)=C(OC)C(OC)=C3)C2)=C1 | Carbonic Anhydrase II and IX | Carbonic Anhydrase II | 2x7u | 1^st^ |
| STX2845 | COC1=C(OC)C=C(C=C1OC)CN2CCC3=CC(OS(=O)(N)=O)=CC=C3C2 | Carbonic Anhydrase II and IX | Carbonic Anhydrase II | 2x7u | 2^nd^ |
| STX2848 | COC1=C(OS(=O)(N)=O)C=C(CC(CC)N(CC2=CC(OC)=C(OC)C(OC)=C2)C3)C3=C1 | Carbonic Anhydrase II and IX | Carbo II | 1g54 | 2^nd^ |
| STX3209 | COC1=C(OS(=O)(N)=O)C=C(CC©N(CC2=CC(OC)=CC=C2Cl)C3)C3=C1 | Carbonic Anhydrase II and IX | Carbo II | 3ml2 | 3^rd^ |
| STX3469 | COC(C(OS(N)(=O)=O)=C1)=CC(CN2CC3=CC=CC(OC)=C3OC)=C1NC2=O | Carbonic Anhydrase II and IX | Carbo II | 1i8z | 3^rd^ |

**Table S8. LigTMap Prediction for the Benchmark Data – HIV**

Source of experimental data: (Pribut *et al.*, 2019)

| **Compound** | **SMILES** | **Expt Target** | **Predicted Target** | **Predicted PDB ID** | **Rank of Prediction** |
| --- | --- | --- | --- | --- | --- |
| **40** | c1ccccc1Oc(ccc2)c(c23)n(c(=O)[nH]3)CC | Non Nucleoside reverse transcriptase | Non Nucleoside reverse transcriptase | 1tv6 | **11^th^** |
| **41** | c1c(C)cc(C)cc1Oc(ccc2)c(c23)n(c(=O)[nH]3)CC | Non Nucleoside reverse transcriptase | Non Nucleoside reverse transcriptase | 2be2 | 1^st^ |
| **42** | N#Cc(c1)cc(Cl)cc1Oc(ccc2)c(c23)n(c(=O)[nH]3)CC | Non Nucleoside reverse transcriptase | Non Nucleoside reverse transcriptase | 3tam | 1^st^ |
| **43** | N#Cc(c1)cc(Cl)cc1Oc(ccc2)c(c23)n(C)c(=O)[nH]3 | Non Nucleoside reverse transcriptase | Non Nucleoside reverse transcriptase | 2jle | 1^st^ |
| **44** | c1c(Cl)cc(C#N)cc1Oc(ccc2)c(c23)n(c(=O)[nH]3)CCC | Non Nucleoside reverse transcriptase | Non Nucleoside reverse transcriptase | 2jle | 1^st^ |
| **45** | N#Cc(c1)cc(C#N)cc1Oc(ccc2)c(c23)n(c(=O)[nH]3)CC | Non Nucleoside reverse transcriptase | Non Nucleoside reverse transcriptase | 2jle | 1^st^ |
| **46** | [nH]1c(=O)n(CC)c(c12)c(ccc2)Oc3cccc(c34)CCCC4 | Non Nucleoside reverse transcriptase | Fail | 1tv6 | 44^th^ |
| **48** | N#Cc(c1)nccc1Oc(ccc2)c(c23)n(c(=O)[nH]3)CC | Non Nucleoside reverse transcriptase | Non Nucleoside reverse transcriptase | 3tam | 7^th^ |
| **49** | n1ccc(C#N)cc1Oc(ccc2)c(c23)n(c(=O)[nH]3)CC | Non Nucleoside reverse transcriptase | Non Nucleoside reverse transcriptase | 3tam | 2^nd^ |
| **50** | N#Cc(c1)cc(/C=C/C#N)cc1Oc(ccc2)c(c23)n(c(=O)[nH]3)CC | Non Nucleoside reverse transcriptase | Non Nucleoside reverse transcriptase | 2jle | 1^st^ |

**Table S9. Activity prediction of HIV non-nucleoside reverse transcriptase inhibitors**

Source of experimental data (Pribut *et al.*, 2019)

| **SMILES** | **Experiment** | **Predicted** | **Diff.** |
| --- | --- | --- | --- |
| c1ccccc1Oc(ccc2)c(c23)n(c(=O)[nH]3)CC | 5.41 | 6.4 | 0.99 |
| c1c(C)cc(C)cc1Oc(ccc2)c(c23)n(c(=O)[nH]3)CC | 6.51 | 7.02 | 0.51 |
| N#Cc(c1)cc(Cl)cc1Oc(ccc2)c(c23)n(c(=O)[nH]3)CC | 7.58 | 7.24 | -0.34 |
| N#Cc(c1)cc(Cl)cc1Oc(ccc2)c(c23)n(C)c(=O)[nH]3 | 7.53 | 7.03 | -0.5 |
| c1c(Cl)cc(C#N)cc1Oc(ccc2)c(c23)n(c(=O)[nH]3)CCC | 7.3 | 7.34 | 0.04 |
| N#Cc(c1)cc(C#N)cc1Oc(ccc2)c(c23)n(c(=O)[nH]3)CC | 7.2 | 7.06 | -0.14 |
| [nH]1c(=O)n(CC)c(c12)c(ccc2)Oc3cccc(c34)CCCC4 | 5.64 | 6.79 | 1.15 |
| N#Cc(c1)nccc1Oc(ccc2)c(c23)n(c(=O)[nH]3)CC | 4.87 | 6.78 | 1.91 |
| n1ccc(C#N)cc1Oc(ccc2)c(c23)n(c(=O)[nH]3)CC | 5.86 | 6.78 | 0.92 |
| N#Cc(c1)cc(/C=C/C#N)cc1Oc(ccc2)c(c23)n(c(=O)[nH]3)CC | 7.3 | 7.15 | -0.15 |

Diff. = Predicted - Experiment

**Table S10. Activity prediction of Ubiquitin ligase HECT inhibitors**

Source of experiment data: (Jessica E Watt *et al.*, 2018)

Diff. = Predicted - Experiment

| SMILES | Experiment | Predicted | Diff. |
| --- | --- | --- | --- |
| Oc1cc(C)c(O)cc1-c2c(O)cc(C)c(c2)O | 6.42 | 5.41 | -1.01 |
| [O-][n+]1ccccc1Sc2ccc([N+](=O)[O-])c(c23)non3 | 6.08 | 5.04 | -1.04 |
| O=C1C=CC(=O)N1C2=CC(=C[C@@H](C2)C(=O)O)N3C(=O)C=CC3=O | 6.07 | 5.21 | -0.86 |
| O=[N+]([O-])c1ccc(c(c12)non2)[S@](=O)c3ccc(C)cc3 | 5.89 | 4.71 | -1.18 |
| c1cccc(c12)n(CCOC)c3c(n2)c(=O)n(c(n3)=O)-c4ccccc4 | 5.64 | 4.8 | -0.84 |

**Table S11. Activity prediction of Tuberculosis inhibitors**

Source of experiment data: (Laura A T Cleghorn *et al.*, 2018)

Diff. = Predicted - Experiment

| **SMILES** | **Experiment** | **Predicted** | **Diff.** |
| --- | --- | --- | --- |
| O[C@@H]([C@H]1O)[C@@H](CO)O[C@H]1N2C3=NC=NC(N4CCN(CC4)C5=CC=CC=C5)=C3N=C2 | 6.92 | 5.32 | -1.60 |
| OC[C@@H]1[C@@H](O)[C@@H](O)[C@H](N2C=NC3=C2N=CC=C3N4CCN(C5=CC=C(C6=CC=CC=C6)C=C5)CC4)O1 | 7.79 | 6.16 | -1.63 |
| OC[C@@H]1[C@@H](O)[C@@H](O)[C@H](N2C=NC3=C2N=CN=C3N4CCN(C5=CC=C(C6=CC=C(OC)N=C6)C=C5)CC4)O1 | 7.73 | 5.86 | -1.87 |
| OC[C@@H]1[C@@H](O)[C@@H](O)[C@H](N2C=NC3=C2N=CN=C3N4CCN(C5=CC=C(C6=CC=C(C(C)(O)C)C=C6)C=C5)CC4)O1 | 7.72 | 6.22 | -1.50 |
| OC[C@@H]1[C@@H](O)[C@@H](O)[C@H](N2C=NC3=C2N=CN=C3C4=CC=C(C#CC5=CC=CC=C5)C=C4)O1 | 7.7 | 5.70 | -2.00 |
| OC[C@@H]1[C@@H](O)[C@@H](O)[C@H](N2C=NC3=C2N=CN=C3N4CCN(C5=CC=C(C6=CC=C(C(F)(F)F)C=C6)C=C5)CC4)O1 | 7.67 | 6.26 | -1.41 |
| OC[C@@H]1[C@@H](O)[C@@H](O)[C@H](N2C=NC3=C2N=CN=C3N4CCN(C5=CC=C(C6=CN(C(F)F)N=C6)C=C5)CC4)O1 | 7.64 | 5.74 | -1.90 |
| OC[C@@H]1[C@@H](O)[C@@H](O)[C@H](N2C=NC3=C2N=CN=C3N4CCN(C5=CC=C(C6=CC=CC(CN7CCOCC7)=C6)C=C5)CC4)O1 | 7.6 | 5.91 | -1.69 |
| OC[C@@H]1[C@@H](O)[C@@H](O)[C@H](N2C=NC3=C2N=CN=C3N4CCN(C5=CC=C(C6=CC=CC=C6)C=C5)CC4)C1 | 7.56 | 6.04 | -1.52 |
| OC[C@@H]1[C@@H](O)[C@@H](O)[C@H](N2C=NC3=C2N=CN=C3N4CCN(C5=CC=C(C6=CC=C(C(F)(F)F)N=C6)C=C5)CC4)O1 | 7.49 | 6.11 | -1.38 |
| OC[C@@H]1[C@@H](O)[C@@H](O)[C@H](N2C=NC3=C2N=CN=C3C#CC4=CC=C(C5=CC=CC=C5)C=C4)O1 | 7.32 | 5.70 | -1.62 |

**Table S12. Activity prediction of Bromodomain inhibitors**

Source of experiment data: (Bamborough *et al.*, 2018)

Diff. = Predicted - Experiment

| **SMILES** | **Experiment** | **Predicted** | **Diff.** |
| --- | --- | --- | --- |
| Cc1cc2ccnc(N[C@H]3CCNC[C@@H]3OCC3CCCCC3)c2[nH]c1=O | 5.6 | 4.1 | 2.25 |
| Cc1cc2c(cnc(N[C@@H]3CCNC[C@H]3OCC3CCCCC3)c2[nH]c1=O)-c1cncc(N)c1 | 6.9 | 6.1 | 0.64 |
| Cc1cncc(c1)-c1cnc(N[C@@H]2CCNC[C@H]2OCC2CCCCC2)c2[nH]c(=O)c(C)cc12 | 6.7 | 6.3 | 0.16 |
| Cc1cncc(c1)-c1cnc(N[C@@H]2CCNC[C@H]2OCC2CCS(=O)(=O)CC2)c2[nH]c(=O)c(C)cc12 | 6.9 | 6.5 | 0.16 |
| CO[C@H]1CNC[C@@H](OCC2CCS(=O)(=O)CC2)[C@@H]1Nc1ncc(-c2cncc(C)c2)c2cc(C)c(=O)[nH]c12 | 6.9 | 6.9 | 0.00 |
| CO[C@H]1CNC[C@@H](OCC2CCC(F)(F)CC2)[C@@H]1Nc1ncc(-c2cncc(C)c2)c2cc(C)c(=O)[nH]c12 | 7.3 | 6.6 | 0.49 |
| CN1CCC(CC1)Nc1nccc2cc(C)c(=O)[nH]c12 | 4.9 | 4 | 0.81 |
| CN1[C@H]2CC[C@@H]1C[C@@H](C2)Nc1nccc2cc(C)c(=O)[nH]c12 | 4.3 | 3.9 | 0.16 |
| CN1[C@H]2CC[C@@H]1C[C@H](C2)Nc1nccc2cc(C)c(=O)[nH]c12 | 4.5 | 3.9 | 0.36 |
| Cc1cncc(c1)-c1cnc(N[C@@H]2CCNC[C@H]2OCC2CCOCC2)c2[nH]c(=O)c(C)cc12 | 6.5 | 5.88 | 0.38 |
| CO[C@H]1CNC[C@@H](OCC2CCOCC2)[C@@H]1Nc1ncc(-c2cncc(C)c2)c2cc(C)c(=O)[nH]c12 | 6.7 | 6.64 | 0.00 |
| Cc1cncc(c1)-c1cnc(N[C@@H]2C[C@@H]3CC[C@@H](N3)[C@H]2CCC2CCOCC2)c2[nH]c(=O)c(C)cc12 | 6.6 | 6.39 | 0.04 |
| Cc1cncc(c1)-c1cnc(N[C@@H]2C[C@H]3CC[C@H](N3)[C@H]2CCC2CCS(=O)(=O)CC2)c2[nH]c(=O)c(C)cc12 | 6.6 | 6.58 | 0.00 |
| Cc1cncc(c1)-c1cnc(N[C@@H]2CCNC[C@H]2OCC2CCC(F)(F)CC2)c2[nH]c(=O)c(C)cc12 | 7.4 | 6.42 | 0.96 |
| Cc1cncc(c1)-c1cnc(N[C@@H]2C[C@@H]3CC[C@@H](N3)[C@H]2CCC2CCC(F)(F)CC2)c2[nH]c(=O)c(C)cc12 | 7 | 6.41 | 0.35 |
| Cc1cncc(c1)-c1ccc(N[C@@H]2C[C@@H]3CC[C@@H](N3)[C@H]2CCC2CCS(=O)(=O)CC2)c2[nH]c(=O)c(C)cc12 | 6.6 | 6.61 | 0.00 |
| Cc1cncc(c1)-c1ccc(N[C@@H]2CCNC[C@H]2OCC2CCS(=O)(=O)CC2)c2[nH]c(=O)c(C)cc12 | 6.8 | 6.62 | 0.03 |
| CO[C@H]1CNCC[C@@H]1Nc1nccc2cc(C)c(=O)[nH]c12 | 5 | 4 | 1.00 |
| CO[C@@H]1CNC[C@H](OC)[C@H]1Nc1nccc2cc(C)c(=O)[nH]c12 | 4.6 | 4.1 | 0.25 |

**Table S13. Activity prediction of Carbonic anhydrase inhibitors**

Source of experiment data**:** (Buemi *et al.*, 2019) (Andring *et al.*, 2019)

Diff. = Predicted - Experiment

| **SMILES** | **Experiment** | **Predicted** | **Diff.** |
| --- | --- | --- | --- |
| O=S(=O)(N)O[C@H]1CC[C@H]([C@@]12C)[C@H]3[C@H](CC2)c4c(CC3)cc(cc4)OS(=O)(=O)N | NA | 7.05 | NA |
| O=S(=O)(N)O[C@H]1CC[C@H]([C@@]12C)[C@H]3[C@H](CC2)c4c(CC3)cc(OS(=O)(=O)N)c(c4)OC | 6.57 | 7.06 | 0.24 |
| O=S(=O)(N)O[C@H]1CC[C@H]([C@@]12C)[C@H]3[C@H](CC2)c4c(CC3)cc(OS(=O)(=O)N)c(c4)CC | 5.62 | 6.81 | 1.42 |
| N#CC[C@H]1CC[C@H]([C@@]12C)[C@H]3[C@H](CC2)c4c(CC3)cc(OS(=O)(=O)N)c(c4)OC | 5.83 | 6.37 | 0.29 |
| O=S(=O)(N)O[C@H]1CC[C@H]([C@@]12C)[C@H]3[C@H](CC2)c4c(CC3)cc(O)c(c4)OC | 4.99 | 6.21 | 1.49 |
| COC1=C(OS(=O)(N)=O)C=C(CCN(C(C2=CC(OC)=C(OC)C=C2)=O)C3=O)C3=C1 | 5.35 | 8.48 | 9.80 |
| OC1=C(OC)C=C2C(CCN(CC3=CC(OC)=C(OC)C(OC)=C3)C2)=C1 | 4.28 | 6.53 | 5.06 |
| COC1=C(OS(=O)(N)=O)C=C(CCN(CC2=CC(OC)=C(OC)C(OC)=C2)C3)C3=C1 | 5.06 | 7.23 | 4.71 |
| OC1=CC=C2C(CCN(CC3=CC(OC)=C(OC)C(OC)=C3)C2)=C1 | 3.17 | 6.53 | 11.29 |
| COC1=C(OC)C=C(C=C1OC)CN2CCC3=CC(OS(=O)(N)=O)=CC=C3C2 | 6.47 | 6.66 | 0.04 |
| COC1=C(OS(=O)(N)=O)C=C(CC(CC)N(CC2=CC(OC)=C(OC)C(OC)=C2)C3)C3=C1 | 4.16 | 7.31 | 9.92 |
| COC1=C(OS(=O)(N)=O)C=C(CC(C)N(CC2=CC(OC)=CC=C2Cl)C3)C3=C1 | 5.27 | 6.71 | 2.07 |
| COC(C(OS(N)(=O)=O)=C1)=CC(CN2CC3=CC=CC(OC)=C3OC)=C1NC2=O | 5.33 | 7.1 | 3.13 |
| O=S(=O)(N)c1ccc(cc1)C(=O)N(CC2)CCC2c3ccccc3 | 9.3 | 7.93 | 1.88 |
| O=S(=O)(N)c1ccc(cc1)C(=O)N(CC2)CCC2(O)c3ccccc3 | 9.22 | 7.58 | 2.69 |
| O=S(=O)(N)c1ccc(cc1)C(=O)N2CCN(CC2)Cc3ccc(F)cc3 | 9.3 | 8.17 | 1.28 |

**Table S14. Activity prediction of Kinase inhibitors**

Source of experiment data (Watts *et al.*, 2019)

Diff. = Predicted - Experiment

| **SMILES** | **Experiment** | **Predicted** | **Diff.** |
| --- | --- | --- | --- |
| O=C1[C@@H](CC)N(C2CCCC2)C3=C(N1C)C=NC(NC4=C(OC)C=C(C5CCN(C)CC5)C=C4)=N3 | 6.54 | 8.08 | 1.54 |
| O=C1[C@@H](CC)N(C2CCCC2)C3=C(N1C)C=NC(NC4=C(OCC(C)C)C=C(C(NC5CCN(C)CC5)=O)C=C4)=N3 | 5.77 | 8.09 | 2.32 |
| O=C1[C@@H](CC)N(C2CCCC2)C3=C(N1C)C=NC(NC4=C(OCC)C=C(C(NC5CCN(C)CC5)=O)C=C4)=N3 | 6.42 | 8.04 | 1.62 |
| O=C1[C@H](CC)N(C2CCCC2)C3=C(N1C)C=NC(NC4=C(OCC)C=C(C(NC5CCN(C)CC5)=O)C=C4)=N3 | 5.47 | 8.05 | 2.58 |
| O=C1CN(C2CCCC2)C3=C(N1C)C=NC(NC4=C(OCC)C=C(C(NC5CCN(C)CC5)=O)C=C4)=N3 | 6.28 | 8.01 | 1.73 |
| O=C1[C@@H](CC)N(CC2=CC(Br)=CC=C2)C3=C(N1C)C=NC(NC4=C(OCC)C=C(C(NC5CCN(C)CC5)=O)C=C4)=N3 | 6.31 | 7.4 | 1.09 |
| O=C1[C@@H](CC)N(CC2=CC=CC=C2)C3=C(N1C)C=NC(NC4=C(OCC)C=C(C(NC5CCN(C)CC5)=O)C=C4)=N3 | 6.36 | 7.64 | 1.28 |
| O=C1[C@@H](CC)N(CC2=CC(Br)=CC=C2)C3=C(N1C)C=NC(NC4=C(OCC)C=C(C5CCN(C)CC5)C=C4)=N3 | 6.54 | 7.51 | 0.97 |
| O=C1[C@@H](CC)N(CC2=CC=CC=C2)C3=C(N1C)C=NC(NC4=C(OCC)C=C(C5CCN(C)CC5)C=C4)=N3 | 7.07 | 7.75 | 0.68 |
| O=C1[C@@H](CC)N(CC2=CC(Cl)=CC=C2)C3=C(N1C)C=NC(NC4=C(OCC)C=C(C5CCN(C)CC5)C=C4)=N3 | 6.68 | 7.79 | 1.11 |
| O=C1[C@@H](CC)N(CC2=CC(C#N)=CC=C2)C3=C(N1C)C=NC(NC4=C(OCC)C=C(C5CCN(C)CC5)C=C4)=N3 | 6.43 | 7.68 | 1.25 |
| O=C1[C@@H](CC)N(CC2=CC(OC)=CC=C2)C3=C(N1C)C=NC(NC4=C(OCC)C=C(C5CCN(C)CC5)C=C4)=N3 | 6.46 | 7.64 | 1.18 |
| O=C1[C@@H](CC)N(CC2=C(Br)C=CC=C2)C3=C(N1C)C=NC(NC4=C(OCC)C=C(C5CCN(C)CC5)C=C4)=N3 | 6.82 | 7.51 | 0.69 |
| O=C1[C@@H](CC)N(CC2=CC=C(Br)C=C2)C3=C(N1C)C=NC(NC4=C(OCC)C=C(C5CCN(C)CC5)C=C4)=N3 | 6.17 | 7.51 | 1.34 |
| O=C1[C@@H](CC)N(CC2=C(C)C=CS2)C3=C(N1C)C=NC(NC4=C(OCC)C=C(C5CCN(C)CC5)C=C4)=N3 | 7.20 | 7.71 | 0.51 |
| O=C1[C@@H](CC)N(CC2=CC(C)=CS2)C3=C(N1C)C=NC(NC4=C(OCC)C=C(C5CCN(C)CC5)C=C4)=N3 | 7.77 | 7.71 | -0.06 |
| O=C1[C@@H](CC)N(CC2=CC=C(C)S2)C3=C(N1C)C=NC(NC4=C(OCC)C=C(C5CCN(C)CC5)C=C4)=N3 | 6.66 | 7.71 | 1.05 |
| O=C1[C@@H](CC)N(CC2=C(C)N=CS2)C3=C(N1C)C=NC(NC4=C(OCC)C=C(C5CCN(C)CC5)C=C4)=N3 | 6.43 | 7.79 | 1.36 |

**(A) Correlation of prediction to experimental ALK activities**

**(B) Correlation of predictions to experimental PLK-1 activities**

| **SMILES** | **Experiment** | **Predicted** | **Diff.** |
| --- | --- | --- | --- |
| O=C1[C@@H](CC)N(C2CCCC2)C3=C(N1C)C=NC(NC4=C(OC)C=C(C5CCN(C)CC5)C=C4)=N3 | 8.004 | 8.08 | 0.08 |
| O=C1[C@@H](CC)N(C2CCCC2)C3=C(N1C)C=NC(NC4=C(OCC)C=C(C(NC5CCN(C)CC5)=O)C=C4)=N3 | 7.921 | 8.04 | 0.12 |
| O=C1[C@@H](CC)N(CC2=CC(Br)=CC=C2)C3=C(N1C)C=NC(NC4=C(OCC)C=C(C(NC5CCN(C)CC5)=O)C=C4)=N3 | 6.959 | 7.4 | 0.44 |
| O=C1[C@@H](CC)N(CC2=CC=CC=C2)C3=C(N1C)C=NC(NC4=C(OCC)C=C(C(NC5CCN(C)CC5)=O)C=C4)=N3 | 7.071 | 7.64 | 0.57 |
| O=C1[C@@H](CC)N(CC2=CC(Br)=CC=C2)C3=C(N1C)C=NC(NC4=C(OCC)C=C(C5CCN(C)CC5)C=C4)=N3 | 6.268 | 7.51 | 1.24 |
| O=C1[C@@H](CC)N(CC2=CC=CC=C2)C3=C(N1C)C=NC(NC4=C(OCC)C=C(C5CCN(C)CC5)C=C4)=N3 | 6.538 | 7.75 | 1.21 |
| O=C1[C@@H](CC)N(CC2=CC(Cl)=CC=C2)C3=C(N1C)C=NC(NC4=C(OCC)C=C(C5CCN(C)CC5)C=C4)=N3 | 6.268 | 7.79 | 1.52 |
| O=C1[C@@H](CC)N(CC2=CC(C#N)=CC=C2)C3=C(N1C)C=NC(NC4=C(OCC)C=C(C5CCN(C)CC5)C=C4)=N3 | 5.745 | 7.68 | 1.94 |
| O=C1[C@@H](CC)N(CC2=C(C)C=CS2)C3=C(N1C)C=NC(NC4=C(OCC)C=C(C5CCN(C)CC5)C=C4)=N3 | 7.167 | 7.71 | 0.54 |
| O=C1[C@@H](CC)N(CC2=CC(C)=CS2)C3=C(N1C)C=NC(NC4=C(OCC)C=C(C5CCN(C)CC5)C=C4)=N3 | 6.903 | 7.71 | 0.81 |

| **Kinase** | **No. of SMILES** | **R** | **RMSE** |
| --- | --- | --- | --- |
| ALK | 18 | -0.33 | 1.11 |
| PLK-1 | 10 | 0.61 | 0.85 |
| **Average of ALK & PLK-1** | **28** | **0.18** | **1.43** |

**(C) Correlation of predictions to experimental PLK-1 activities**

**Table S15. Activity prediction of Beta secretase inhibitors**

Source of experiment data (Fujimoto *et al.*, 2019)

Diff. = Predicted - Experiment

| **SMILES** | **Experiment** | **Predicted** | **Diff.** |
| --- | --- | --- | --- |
| C[C@]1(CS(N(C)C(N)=N1)(=O)=O)C2=CC(NC(C3=NC=C(F)C=C3)=O)=CC=C2F | 8.72 | 8.03 | 0.48 |
| NC1=N[C@]([C@]2([H])CS1)(CO[C@@H]2C)C3=CC(NC(C4=NC=C(C(F)F)N=C4)=O)=CC=C3F | 8.41 | 8.11 | 0.09 |
| NC1=N[C@]2(C3=C(C[C@@]42CC[C@@H](OC)CC4)C=CC(C5=CN=CC(C#CC)=C5)=C3)N=C1C | 8.92 | 8.03 | 0.80 |
| NC1=N[C@@](C2=CC(C3=CN=CN=C3)=CC=C2)(C4=CC(CC)=NC(CC)=C4)C(N1C)=O | 7.36 | 7.91 | 0.30 |
| FC1=CC=C(NC(C2=NC=C(Cl)C=C2)=O)C=C1[C@]3(C)CCSC(N)=N3 | 8.34 | 7.73 | 0.38 |
| FC1=CC=C(NC(C2=NC=C(F)C=C2)=O)C=C1[C@]3(C)CCSC(N)=N3 | 7.89 | 7.77 | 0.02 |
| FC1=CC=C(NC(C2=NC=C(C#N)C=C2)=O)C=C1[C@]3(C)CCSC(N)=N3 | 8.49 | 7.76 | 0.53 |
| FC1=CC=C(NC(C2=NC=C(C(F)F)N=C2)=O)C=C1[C@]3(C)CCSC(N)=N3 | 7.7 | 7.97 | 0.08 |
| FC1=CC=C(NC(C2=NC=C(OC)N=C2)=O)C=C1[C@]3(C)CCSC(N)=N3 | 7.96 | 7.89 | 0.00 |
| FC1=CC=C(NC(C2=NC=C(OCC(F)(F)F)N=C2)=O)C=C1[C@]3(C)CCSC(N)=N3 | 8.14 | 7.78 | 0.13 |
| FC1=CC=C(NC(C2=NC=C(OCF)N=C2)=O)C=C1[C@]3(C)CCSC(N)=N3 | 8.31 | 7.78 | 0.28 |
| FC1=CC=C(NC(C2=NC=C(OCF)N=C2)=O)C=C1[C@]3(C)[C@@H](OC)CSC(N)=N3 | 8.08 | 8.16 | 0.01 |
| FC1=CC=C(NC(C2=NC=C(OCF)N=C2)=O)C=C1[C@]3(C)[C@@H](C4=CC=CC=C4)CSC(N)=N3 | 8.77 | 7.97 | 0.63 |
| FC1=CC=C(NC(C2=NC=C(OCF)N=C2)=O)C=C1[C@]3(C)[C@H](C4=CC=CC=C4)CSC(N)=N3 | 8.48 | 7.97 | 0.26 |
| FC1=CC=C(NC(C2=NC=C(OCF)N=C2)=O)C=C1[C@]3(C)[C@@H](C4CCOCC4)CSC(N)=N3 | 8.49 | 8.18 | 0.09 |
| FC1=CC=C(NC(C2=NC=C(OCF)N=C2)=O)C=C1[C@]3(C)C4(CCOCC4)CSC(N)=N3 | 7.82 | 7.79 | 0.00 |
| FC1=CC=C(NC(C2=NC=C(OCF)N=C2)=O)C=C1[C@]3(C)C4(CC(F)(F)C4)CSC(N)=N3 | 8.26 | 7.77 | 0.24 |
| FC1=CC=C(NC(C2=NC=C(OCF)N=C2)=O)C=C1[C@]3(C)C4(CCC(F)(F)CC4)CSC(N)=N3 | 7.8 | 7.77 | 0.00 |
| FC1=CC=C(NC(C2=NC=C(OCF)N=C2)=O)C=C1[C@]3(C)C4(CCS(CC4)(=O)=O)CSC(N)=N3 | 8.42 | 7.93 | 0.24 |

**References**

1. Andring,J.T. *et al.* (2019) 3,17β-Bis-sulfamoyloxy-2-methoxyestra-1,3,5(10)-triene and Nonsteroidal Sulfamate Derivatives Inhibit Carbonic Anhydrase IX: Structure-Activity Optimization for Isoform Selectivity. *J. Med. Chem.*, **5**.
2. Bamborough,P. *et al.* (2018) Aiming to Miss a Moving Target: Bromo and Extra Terminal Domain (BET) Selectivity in Constrained ATAD2 Inhibitors. *J. Med. Chem.*, **61**, 8321–8336.
3. Berman,H.M. *et al.* (2000) The Protein Data Bank. *Nucleic Acids Res.*, **28**, 235–242.
4. Buemi,M.R. *et al.* (2019) Exploring structural properties of potent human carbonic anhydrase inhibitors bearing a 4-(cycloalkylamino-1-carbonyl)benzenesulfonamide moiety. *Eur. J. Med. Chem.*, **163**, 443–452.
5. Cleghorn,Laura A T *et al.* (2018) Identification of Morpholino Thiophenes as Novel Mycobacterium tuberculosis Inhibitors, Targeting QcrB. *J. Med. Chem.*, **61**, 6592–6608.
6. Cleghorn,Laura A.T. *et al.* (2018) Identification of Morpholino Thiophenes as Novel Mycobacterium tuberculosis Inhibitors, Targeting QcrB. *J. Med. Chem.*, **61**, 6592–6608.
7. Fujimoto,K. *et al.* (2019) Structure-Based Design of Selective β-Site Amyloid Precursor Protein Cleaving Enzyme 1 (BACE1) Inhibitors: Targeting the Flap to Gain Selectivity over BACE2. *J. Med. Chem.*, **62**, 5080–5095.
8. Morris,G.M. *et al.* (2009) AutoDock4 and AutoDockTools4: Automated docking with selective receptor flexibility. *J. Comput. Chem.*, **30**, 2785–2791.
9. Pribut,N. *et al.* (2019) Aryl Substituted Benzimidazolones as Potent HIV-1 Non-Nucleoside Reverse Transcriptase Inhibitors. *ACS Med. Chem. Lett.*, **10**, 196–202.
10. RDKit: Open-source cheminformatics.
11. Schrödinger Release 2017-4 (2017).
12. Tai,H.K. *et al.* (2018) Chaos-embedded particle swarm optimization approach for protein-ligand docking and virtual screening. *J. Cheminform.*, **10**, 1–13.
13. Wang,L. *et al.* (2018) Discovery of novel pyrrolopyrimidine/pyrazolopyrimidine derivatives bearing 1,2,3-triazole moiety as c-Met kinase inhibitors. *Chem. Biol. Drug Des.*, **92**, 1301–1314.
14. Wang,R. *et al.* (2004) The PDBbind database: collection of binding affinities for protein-ligand complexes with known three-dimensional structures. *J. Med. Chem.*, **47**, 2977–80.
15. Watt,Jessica E *et al.* (2018) Discovery of Small Molecule WWP2 Ubiquitin Ligase Inhibitors. *Chemistry*, **24**, 17677–17680.
16. Watts,E. *et al.* (2019) Designing Dual Inhibitors of Anaplastic Lymphoma Kinase (ALK) and Bromodomain-4 (BRD4) by Tuning Kinase Selectivity. *J. Med. Chem.*, **62**, 2618–2637.
17. Wójcikowski,M. *et al.* (2015) Open Drug Discovery Toolkit (ODDT): A new open-source player in the drug discovery field. *J. Cheminform.*, **7**, 1–6.
